# Biochemical and Structural Insights into the Mechanism of DNA Recognition by Arabidopsis ETHYLENE INSENSITIVE3

**DOI:** 10.1371/journal.pone.0137439

**Published:** 2015-09-09

**Authors:** Jinghui Song, Chenxu Zhu, Xing Zhang, Xing Wen, Lulu Liu, Jinying Peng, Hongwei Guo, Chengqi Yi

**Affiliations:** 1 State Key Laboratory of Protein and Plant Gene Research, School of Life Sciences, and Peking-Tsinghua Center for Life Sciences, Peking University, Beijing, China; 2 Department of Chemical Biology and Synthetic and Functional Biomolecules Center, College of Chemistry and Molecular Engineering, Peking University, Beijing, China; Institute of Genetics and Developmental Biology, Chinese Academy of Sciences, CHINA

## Abstract

Gaseous hormone ethylene regulates numerous stress responses and developmental adaptations in plants by controlling gene expression via transcription factors ETHYLENE INSENSITIVE3 (EIN3) and EIN3-Like1 (EIL1). However, our knowledge regarding to the accurate definition of DNA-binding domains (DBDs) within EIN3 and also the mechanism of specific DNA recognition by EIN3 is limited. Here, we identify EIN3 82–352 and 174–306 as the optimal and core DBDs, respectively. Results from systematic biochemical analyses reveal that both the number of EIN3-binding sites (EBSs) and the spacing length between two EBSs affect the binding affinity of EIN3; accordingly, a new DNA probe which has higher affinity with EIN3 than *ERF1* is also designed. Furthermore, we show that palindromic repeat sequences in *ERF1* promoter are not necessary for EIN3 binding. Finally, we provide, to our knowledge, the first crystal structure of EIN3 core DBD, which contains amino acid residues essential for DNA binding and signaling. Collectively, these data suggest the detailed mechanism of DNA recognition by EIN3 and provide an in-depth view at molecular level for the transcriptional regulation mediated by EIN3.

## Introduction

The plant hormone ethylene regulates numerous growth and developmental processes, including inhibition of cell expansion, regulation of seed germination, promotion of leaf and flower senescence, induction of fruit ripening and abscission, and response to pathogens and stress [1–5]. A largely putative ethylene signaling pathway from hormone perception at the endoplasmic reticulum membrane to transcriptional regulation in the nucleus was proposed based on genetic and molecular studies [6–8]. Briefly, ethylene is perceived by a five-member receptor family including ETR1, ETR2, ERS1, ERS2, and EIN4 [9–14]. In the absence of ethylene, the receptors constitutively activate a negative regulator CTR1, which acts upstream of the positive regulator EIN2 [15–17]. Downstream of EIN2, the signal is transmitted to two necessary transcription factors ETHYLENE INSENSITIVE3 (EIN3) and EIN3-Like1 (EIL1) by protecting them from EIN3-BINDING F-BOX PROTEIN 1/2 (EBF1/2)-mediated ubiquitin-proteasome degradation; thus, function-complementary EIN3 and EIL1 confer specificity to ethylene response through selection of target genes in the nucleus [18–24].

**Fig 1:**
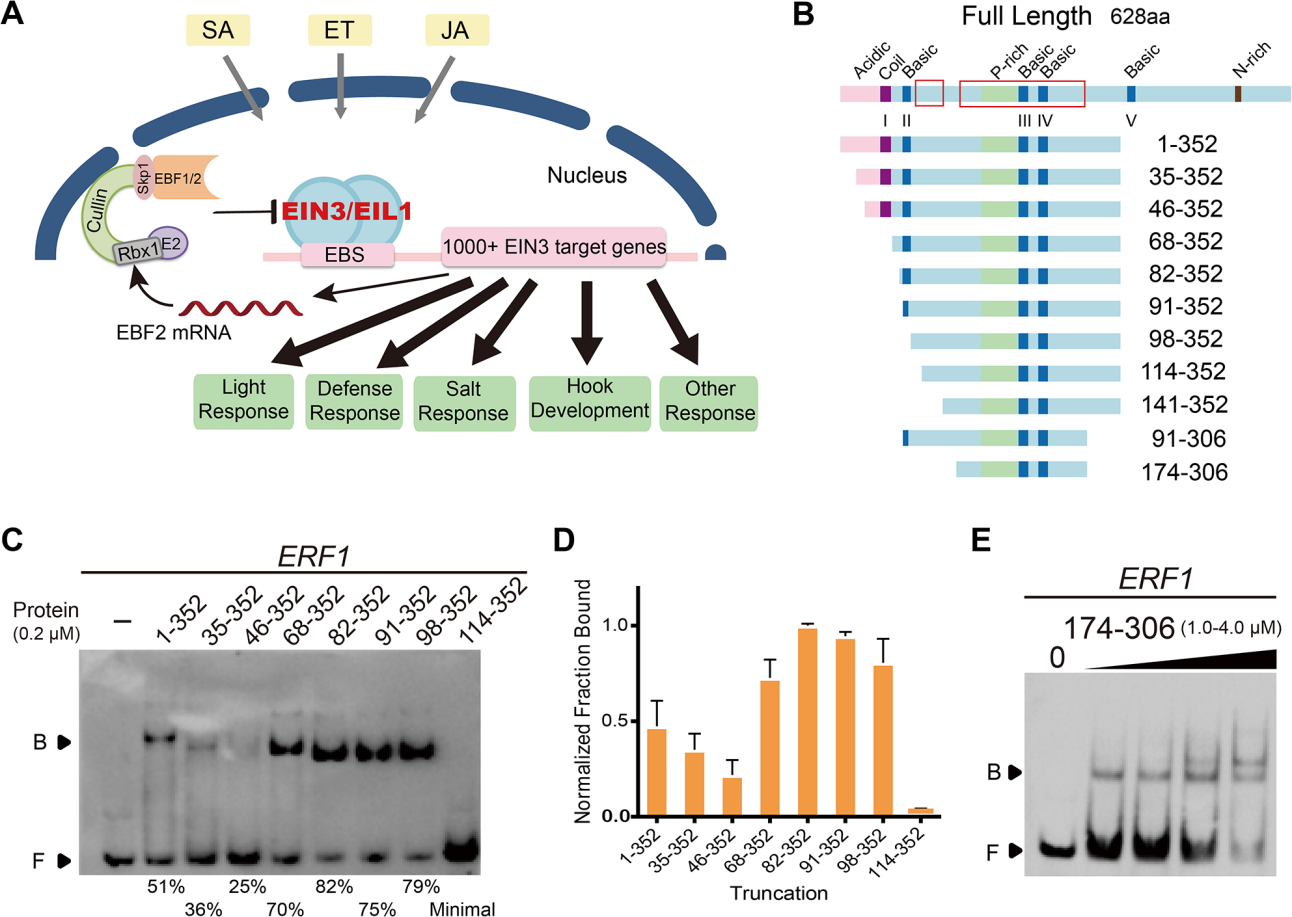
The optimal and core DNA-binding domains of EIN3. (A) A Schematic model for EIN3 action in multiple signaling pathways. (B) Schematic structural diagram of full length and truncated EIN3 proteins. Acidic region, coil region, proline-rich region, basic domains, and asparagine-rich domain are represented by different color filled boxes. Highly conserved regions are also indicated by red boxes. (C) Representative EMSA results of different EIN3 truncations binding to a 3’–biotin labeled *ERF1* probe. Specific EIN3 truncations used for each lane are shown in the upper panel and the fraction bound is shown in the lower panel. EIN3 114–352 displayed minimal binding under the current protein concentration, yet clearly bound to DNA at higher concentration (S3C Fig). In addition, the same amount of different EIN3 truncations was used in each PAGE-gel in order to have better visual comparisons, and similar results were obtained at other concentrations of proteins (S3A Fig). “B”: bound; “F”: free. (D) Relative binding data for 0.2 μM truncated EIN3 proteins with *ERF1*. The data were normalized to the fraction bound of 82–352 with *ERF1*; error bars represented the standard deviation from three independent experiments. (E) EMSA results of the core DBD (174–306), which retained the ability to bind to *ERF1*.

EIN3/EIL1 are the critical transcription factors in the ethylene-initiated signaling network. The Ecker Group first identified the loss of ethylene-mediated effects in mutants of the *Arabidopsis* EIN3 gene [25]. In ethylene signaling pathway, EIN3 regulates the expression of its direct target genes, such as *ETHYLENE RESPONSE FACTOR1* (*ERF1)* and *HOOKLESS1 (HLS1)* [24, 26]. In addition to its essential roles in the ethylene signaling pathway, EIN3 also participated in the crosstalk among different hormonal pathways (Fig 1A). We previously revealed that EIN3/EIL1 mediate jasmonate-ethylene (JA-ET) signaling synergy in defense against necrotrophic pathogens [27], while EIN3 also plays a key role in the JA-ET antagonism in apical hook development by interacting with MYC2 (JA-activated transcription factor) [28, 29]. In the transition from skotomorphogenesis to photomorphogenesis, EIN3/EIL1 cooperate with PIF1 to prevent photo-oxidative damage and optimize de-etiolation of *Arabidopsis* seedlings, and *protochlorophyllide oxidoreductase A* and *B (PORA/B)* are direct target genes of EIN3 [30, 31]. In plant innate immunity, EIN3/EIL1 directly repress the *SALICYLIC ACID INDUCTION DEFICIENT2* (*SID2)* expression to decrease salicylic acid (SA) level and downregulate pathogen/microbe-associated molecular patterns (PAMP) defenses, while EIN3 (potentially EIL1) direct induces *FLS2* expression to promote PAMP defenses [32, 33]. Furthermore, EIN3 directly targets *ESE1 (At3g23220)*, and ESE1 regulates salt-related genes [34]. We also found that *EIN3* is a senescence-associated gene that advances the progression of age-dependent leaf senescence [35]. Moreover, EIN3 is not only degraded through EBF1/2-dependent proteolysis, but also modulates *EBF2* gene expression via the elaborate feedback regulation [36]. In all, EIN3/EIL1 act as a signaling hub that integrates the plant signals to adapt complex environment.

As a highly important transcription factor, one crucial function of EIN3 is to recognize and bind to specific target genes. The Ecker group first showed that EIN3/EILs family proteins regulate gene expression by binding directly to a primary ethylene response element (17); recently, they further identified the repertoire of EIN3 target genes, using ChIP-Seq and mRNA-Seq during a timecourse of ethylene treatment [26]. However, a central, yet unanswered question is how EIN3 recognizes and binds to a wide variety of target DNA. Previous studies have demonstrated that the N-terminal region of EIN3 (for instance, residues 1–359, 1–314, 141–352 used by different studies, and also its homolog in tobacco TEIL 82–302) is responsible for DNA binding [24, 30, 32, 37], while the optimal DNA-binding domain (DBD) and the core DBD of EIN3 are still unknown. In addition, EIN3 was shown to bind specifically to the fragment of *ERF1* promoter (−1213 to −1178) which has two palindromic repeats flanking one central EIN3-binding site (EBS) [24]; yet except for ERF1, no other EIN3 target bearing palindromic repeat sequences has been reported. Hence whether or not such palindromic sequence is necessary for DNA binding is unclear. Furthermore, although the Yamasaki et al. have reported the solution structure of EIL3 major DBD [38], EIL3 subgroup are distinct from other EIL family members and may have functions in regulating sulfur responses instead of mediating ethylene responses [39]. Thus, a crystal structure of EIN3 would still be desired for the inspection–at atomic level–of residues essential to the function of EIN3 [25, 32, 40]. Consequently, such limited knowledge regarding to the molecular mechanism of the interaction between EIN3 and DNA targets still significantly impede an in-depth view of transcriptional regulation initiated by EIN3.

To better understand the interactions between EIN3 and its DNA targets, we herein perform systematic and detailed biochemical studies and present, to our knowledge, the first crystal structure of EIN3. We identify the optimal and core DBDs of EIN3, and show that both the number of EBSs and the length of spacing between two EBSs have great impact on DNA-binding preference. Furthermore, we show that the palindromic repeats in *ERF1* are not necessary for DNA binding. Finally, a 1.78 Å crystal structure of the core DBD of EIN3 [Protein Data Bank (PDB) accession number: 4ZDS] reveals the novel folding of DBD for the EIN3/EIL family proteins and offers insights into the mechanistic details of key amino acid residues involved in the biological functions of EIN3.

## Materials and Methods

### Protein expression and purification

Different truncations to the DNA-binding domain (DBD) of Arabidopsis EIN3 (At3g20770; residues 1–352, 35–352, 46–352, 68–352, 82–352, 91–352, 98–352, 114–352, 141–352, 91–306 and 174–306) were PCR-amplified from cDNA clones and inserted into the NheI and BamHI restriction sites of the expression vector pET-28a (Novagen), generating N-terminal hexahistidine-tagged constructs. The P216S mutation was introduced using the Fast Mutagenesis System (Beijing TransGen Biotech Co., Ltd) on the plasmid of EIN3 82–352. All clones were confirmed by Sanger sequencing.

For protein expression, Transetta (DE3) competent cells (Beijing TransGen Biotech Co., Ltd) were transformed with the corresponding vectors described above. Cells were grown at 37°C in LB medium with 50 μg/ml kanamycin. Protein expression was induced at 20°C when the culture reached an OD_600_ of 0.6 by the addition of isopropyl-β-D-1-thio-galactopyranoside to a final concentration of 0.1 mM and cells were allowed to grow for 16 additional hours. For seleno-methionine (SeMet) derivative of EIN3, cells were grown overnight at 37°C in LB medium, and diluted twice at an 1:100 ratio in M9 medium supplemented with amino acids (L-Lys, L-Phe and L-Thr at 100 mg/l; L-Iso, L-Leu and L-Val at 50 mg/l) and 50 mg/l L-Selenomethionine, and further allowed to grow to OD_600_ of 0.5. Next, expression of protein was induced similarly as described above.

Cells were then harvested by centrifugation at 4,000g for 35 min and resuspended in lysis buffer (100 mM Na_2_HPO_4_/NaH_2_PO_4_, pH 6.5, 300 mM NaCl, 2 mM DTT and 1 mM PMSF). All subsequent steps were conducted either on ice or at 4°C. Cells were lysed by sonication and cleared by centrifugation for 30 min at 15,000g. The supernatant was purified by HiLoad HisTrap (Ni-NTA) column (GE Healthcare) and Superdex 75 PG size-exclusion column (GE Healthcare) equilibrated in 40 mM Na_2_HPO_4_/NaH_2_PO_4_, pH 6.5, 90 mM NaCl, and 1 mM DTT, using an ÄKTA pure system (GE Healthcare). Peak fractions were analyzed by SDS-PAGE, pooled, concentrated, flash frozen in liquid nitrogen, and stored at − 80°C. Protein concentrations were determined from UV absorbance at 280 nm by 8453 UV-Vis (Agilent). Numbering of EIN3 residues is based on UniProt accession number O24606.

Size exclusion chromatography of purified EIN3 82–352 protein under different salt concentration were performed on Superdex 75 PG size-exclusion column (GE Healthcare) using an ÄKTA pure system (GE Healthcare). Superdex 75 PG size-exclusion column (GE Healthcare) was equilibrated in 10 mM Tris–HCl, pH 7.5, 1 mM DTT, and three different NaCl concentrations (100 mM/300 mM/500 mM), respectively. All steps were conducted at 4°C.

### Mass spectrometry

Purified EIN3 82–352 protein was run on a 12% SDS-PAGE gel and the band corresponding to EIN3 was sliced. Protein was then digested with trypsin and analyzed using Liquid chromatography-tandem mass spectrometry of the Analytical Instrumentation Center (Peking University).

### Oligonucleotides

All DNA oligonucleotides were synthesized and further purified with HPLC by the Shanghai Sangon Biotechnology Company (Shanghai, China). DNA concentrations were determined from UV absorbance at 260 nm by 8453 UV-Vis (Agilent). The forward strands were labeled with the Biotin 3’ End DNA Labeling Kit (Pierce). The DNA duplexes used for EMSA experiments were generated by heating the mixture of complementary oligonucleotides at 95°C for 5 min and slowly cooling to 20°C over 2h in the annealing buffer of 10 mM Tris–HCl (pH 7.5), 100 mM NaCl. All oligonucleotide sequences are listed in S1 Table.

The annealed probes were further purified using Thermo DNAPac PA 200 RS column on Agilent 1260 HPLC system [with a NaCl gradient of 0.4–1.0M in 10 mM Tris–HCl (pH 7.5) buffer over the course of 12.5 min]. As the NaCl gradient was applied, *2EBS-S10* reverse strand was first eluted, followed by forward strand and finally the desired *2EBS-S10* dsDNA probe. The corresponding fractions were collected and the buffer was exchanged to the annealing buffer using Bio-Rad Micro Bio-Spin P-6 Gel Columns. Denaturing PAGE and Native PAGE were used to validate the purified single-stranded and double-stranded probes. Next, EMSA experiments were performed for *K*_d_ determination using both probes before and after purification (so as to compare the probes). The *K*_d_ values for the two probes were very close (deviations were less than 5%). To further check the efficiency of the annealing process, we analyzed the quality and purity of the resulting double-stranded probes. The result showed the content of annealed dsDNA probe was ~95%. Furthermore, the EIN3 truncation 82–352 failed to display noticeable binding to ssDNA probes. Therefore, double-stranded probes in the following EMSA experiments were used directly after annealing without further purification.

### Electrophoretic mobility shift assays

EMSA was performed using the LightShift Chemiluminescent EMSA Kit (Pierce). A typical binding reaction was performed in a total volume of 20 μL by incubation of an appropriate amount of purified EIN3 protein with 1 nM biotin-labeled DNA probe, in 1X binding buffer [10 mM Tris–HCl, pH 7.5, 40 mM KCl, 3 mM MgCl_2_, 1 mM EDTA, 10% glycerol, 1 mM DTT, 1 μg poly(dI-dC)] at room temperature for 30 min. The binding products were resolved on 6% polyacrylamide gel run in 0.5 X Tris-borate-EDTA. Although the absolute amount of probes in EMSA binding systems (20 μl) were the same for different probes (as determined by 8453 UV-Vis), the labeling efficiency may vary for each probe, and hence the intensity on the gel. Signal intensities of bands on the gel were visualized using ChemiDoc MP Imaging System (BIO-RAD).Therefore, DNA binding of EIN3 to different probes was determined by the percentage of the shifted bands, fraction bound, in the equation below. Signal intensities of bands were measured by Image Lab Software (Bio-Rad).

$$\text{Fraction bound}=\frac{{\text{Signal}}_{\text{bound}}}{{\text{Signal}}_{\text{bound}}+{\text{Signal}}_{\text{free}}}\times 100\%$$

### Circular dichroism analysis

Circular dichroism (CD) measurements of purified recombinant 82–352 and 174–306 were performed on a Chirascan spectropolarimeter (Applied Photophysics, Leatherhead, UK). Protein samples were prepared in 10 mM PBS pH 7.0 at a concentration of ~0.2 mg/ml. Spectra were recorded from 190 to 250 nm. The software CDNN[41] was used to calculate alpha-helix content from CD data.

### Size exclusion chromatography with multi-angle light scattering and dynamic light scattering

EIN3 truncations were run at 1 ml/min in 10 mM Tris–HCl, pH 7.5, 100 mM NaCl, and 1 mM DTT on a Superdex 200 analytical size-exclusion column (GE Healthcare) attached to a light scattering diode array (Dawn Heleos II, Wyatt Technology, UK) and dynamic light scattering instrument (DynaPro NanoStar, Wyatt Technology, UK). This allows for the accurate measurement of protein molecular mass without bias from its shape by calculating the ratio of light scattering to the differential refractive index of a sample. Additionally, the DLS can measure the sizes of macromolecules as hydrodynamic radius (Rh). The reproducibility of different runs was assessed using a BSA standard before and after each experiment to see if there was variability in either calculated molecular weight or elution volume. No detectable change was observed over these experiments. All steps were conducted at 4°C.

Size exclusion chromatography with multi-angle static light scattering (SEC-MALS) experiments of EIN3 82–352 were also performed with an Agilent ProSEC 300S column of 5 μm particle size and a 7.5 X 300 mm column size on Agilent 1260 Infinity Multi-Detector GPC/SEC System at 1 ml/min in low salt buffer (10 mM Tris–HCl, pH 7.5, 100 mM NaCl, and 1 mM DTT) and high salt buffer (10 mM Tris–HCl, pH 7.5, 300 mM NaCl, and 1 mM DTT), respectively. The samples were preserved at 4°C before injection; the SEC and detector were performed at room temperature.

### Protoplast Transfection Assay

The constructs containing 36 bp *1EBS-S10-36*, *2EBS-S10-36* or *ERF1* sequence respectively in the pGreenII 0800-LUC vector were used as reporter plasmids [42]. The *renilla luciferase (REN)* gene under the control of 35S promoter in the pGreenII 0800-LUC vector was used as internal control. The construct containing coding sequence of EIN3 in pGreen II 62-SK vector was used as effector plasmid [42]. All clones were confirmed by Sanger sequencing. The sequences of 36 bp *1EBS-S10-36*, *2EBS-S10-36* and *ERF1* used in transient transcriptional activity assay are listed in S1 Table. *Arabidopsis* mesophyll protoplast was prepared as described [43]. 5 μg of reporter plasmid together with 10 μg of indicated effector plasmids was cotransformed into the protoplast. The Dual-Luciferase Reporter Assay System (Promega) was used to measure the LUC and REN activities after 14 hour incubation. The activity of *LUC* and *REN* are measured sequentially from a single sample on a GLO-MAX 20/20 luminometer (Promega). The ratio of LUC/REN is calculated to indicate the final transcriptional activity. The Relative Fluorescence LUC/REN represents the relative LUC/REN ratio normalized by the control group. Data are means (±SD) of three biological replicates.

### Crystal growth and data collection

174–306 SeMet derivative and native crystals were grown from a 1+1 μl sitting drop of, respectively, 10 mg/ml protein solution and crystallization buffer (0.1 M HEPES, pH 7.5, 25% w/v Polyethylene glycol 3,350). Needle-shaped crystals grew to their maximum size within one week and were immediately frozen in crystallization buffer supplemented with 20% glycerol. Diffraction data were collected at 100K at the Shanghai Synchrotron Research Facility beamline BL-17U, China.

### Structure determination and refinement

All diffraction data were processed using HKL2000 program suite [44]. The initial structure was solved using the PHENIX AutoBuild [45] using SAD data measured at the Se K absorption edge on SeMet-EIN3. Native structure was solved by molecular replacement with SeMet-EIN3 as model structure using PhaserMR [46]. Refinement was performed using REFMAC [47, 48] of the CCP4 program suite [49]. Visualization and manual adjustments were done using Coot [50]. Molecular graphics figures were generated using PyMOL (PyMOL Molecular Graphics system, Version 1.3 Schrodinger, LLC). Atomic coordinates and structure factors of EIN3 were deposited in the PDB with the code 4ZDS. A full summary of the data processing and refinement statistics is shown in Table 1.

**Table 1:** Data collection and refinement statistics.

	SeMet-EIN3	Native-EIN3
**Space group**	P1	P1
**Unit cell**	34.82, 44.65, 47.43, 89.19, 77.21, 77.34	35.02, 45.17, 47.57, 89.46, 77.07, 71.38
**Resolution (Å)**	50–2.25 (2.29–2.25)	50.00–1.78 (1.81–1.78)
***R***_**sym**_ **or *R***_**merge**_	0.104 (0.605)	0.053 (0.698)
***I* /σ**	8.6 (2.1)	22.8 (1.9)
**Completeness (%)**	90.1 (95.3)	95.8 (97.0)
**Redundancy**	1.9 (1.9)	3.9 (3.9)
**Resolution (Å)**	46.17–2.24	46.26–1.78
**No. reflections**	11409	23591
***R***_**work**_ **/ *R***_**free**_	19.6/24.2	17.6/21.8
**No. atoms**	2042	2250
**Protein**	173–302; 174–303	173–304; 173–301
**Water atoms**	13	141
**Ramachandran Favored (%)**	96.12	98.44
**Ramachandran Allowed (%)**	2.71	0.78
**Ramachandran Disallowed (%)**	1.16	0.78
**RMS (bonds)**	0.016	0.018
**RMS (angles)**	1.872	2.054
**PDB ID**		4ZDS

## Results

### Identification of the optimal and core DNA-binding domains of EIN3

The N-terminus of EIN3 contains five so-called “basic domains” (BD I: 53–66, BD II: 88–94, BD III: 238–248, BD IV: 265–274 and BD V: 378–384), each of which having 5–8 Lys or Arg residues (Fig 1B) [25]; it also has a proline-rich region (199–240) and such region can be important among many ligands for protein-protein interactions [51]. The more diverse C-terminal half of EIN3 is believed to involve in protein–protein interactions [18].

Previous studies have established the highly conserved N-terminal regions of EIN3 (for instance, residues 1–359, 1–314, 141–352 and its homolog in tobacco, TEIL 82–302) to be competent for DNA binding [24, 30, 32, 37]. However, the exact location of EIN3 DBD was unclear from its primary structure, since the EIN3/EIL proteins show no sequence similarity to other known DBDs. Therefore, we first performed systematic comparisons among different truncations of EIN3 for accurate definition of its DBD. To explore the optimal and core DBDs of EIN3, we made a series of EIN3 truncations with gradually truncated N- and C-terminus, based on secondary structure prediction of EIN3 [using the PSIPRED[52] server] (Fig 1B, S2 Fig and S1 File). We chose a probe originating from the promoter sequence of *ERF1* (-1213 to -1178), a well-established EIN3 target, for electrophoresis mobility-shift assay (EMSA). The results showed that EIN3 1–352 was capable of binding to the *ERF1* probe, consistent with previous experiments using EIN3 1–359 (Fig 1C and 1D; S3A Fig) [24]. The fraction bound decreased from EIN3 1–352 to 35–352 and 46–352, with the proteins expressed and purified to equally-well degree. Surprisingly, as the N-terminus was further truncated, (to residues 68, 82, 91 and 98), DNA-binding ability was fully restored. In fact, 82–352 exhibits the strongest DNA-binding ability among all truncations tested, even stronger than the longer form 1–352, which has an intact N-terminus. Further deletion of the N-terminal region to residue 114, 141 and 174 caused a sharp decline in DNA-binding ability, and shortening of the C-terminus to residue 306 also decreased DNA binding (Fig 1E and S3 Fig). Therefore, basic domains BD III, BD IV, and the proline-rich region in EIN3 are necessary for DNA binding, while the immediate C-terminal region of BD II and region between BD IV and BD V can further enhance the DNA–binding capacity.

Among the 11 EIN3 truncations we tested, 82–352 contains the most conserved sequences within the EIN3/EIL1 family and has the highest DNA-binding affinity; 174–306 contains the entire continuous conserved amino acid residues in the DBD and also has the ability to bind DNA (Fig 1 and S3 Fig). Therefore, we define EIN3 82–352 and 174–306 as the optimal DBD and the core DBD of EIN3, respectively. Furthermore, alpha-helix was the major secondary structure for the optimal and core DBDs, with a higher proportion for the latter truncation determined by circular dichroism (CD) spectrum analysis (S3F Fig), implying that the alpha-helix structure was important for EIN3 to bind to its target DNAs. As the optimal DBD of EIN3 has the highest DNA-binding affinity, all experiments below refer to EIN3 82–352 unless otherwise stated.

### Specific inverted repeat sequences within DNA share higher affinity with EIN3

**Fig 2:**
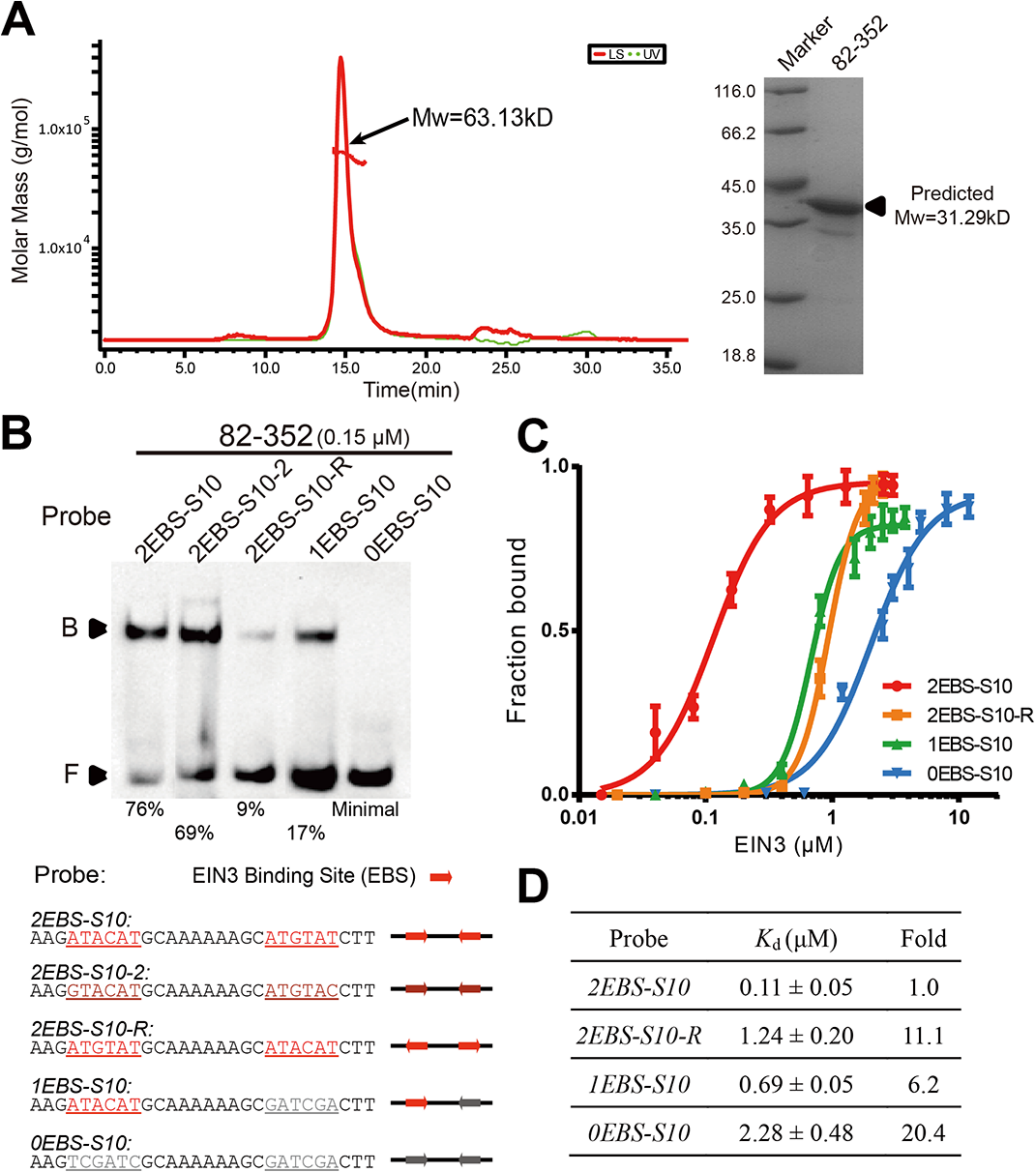
Specific inverted repeat sequences within DNA share higher affinity with EIN3. (A) Analysis of EIN3 82–352 by SEC-MALS and SDS-PAGE. Measured molecular weight (Mw) of EIN3 82–352 was 63.13 kDa (± 0.474%), suggesting EIN3 existed as a homodimer in solution (Predicted Mw: monomer = 31.29 kDa; dimer = 62.58 kDa). Nevertheless, in SDS-PAGE a band corresponding to monomer was observed. (B) EIN3 binding to a series of DNA probes with varied EBS sequence. The sequences of DNA probes are listed in the lower panel. The (dark) red arrows indicate the position and direction of reported EBSs while the grey arrows represent the mutated EBSs. The names of different probes are given so as to reflect the number of EBS sequences, the length of spacing in between, and the relative orientation of EBSs, if possible. For example, probe “*2EBS-S10*” contains two inverted EBSs, which is separated by a 10 bp spacing. The fraction bound is shown right under the gel image. (C) Quantified binding data for 4 probes from (B) fitted with specific binding with Hill slope of GraphPad Prism 6.0. (D) Dissociation constants (*K*_d_) for different probes. Results were obtained from three independent replicates, and the relative folds were normalized to the *K*_d_ of *2EBS-S10*.

It has been shown that EIN3 binds its DNA target as a homodimer [24]. However, whether or not EIN3 can form a dimer in the absence of DNA is not known. To address this question, we performed size exclusion chromatography with multi-angle static light scattering (SEC-MALS) analysis and measured the molecular weight (Mw) of EIN3 82–352. MALS results showed that EIN3 82–352 (predicted Mw: ~31.3 kDa) had a measured Mw of ~63.1 kDa in solution, consistent with a dimer that was independent of DNA binding (Fig 2A). This dimer is also stable under higher salt concentrations (S4 Fig). In addition, EIN3 was a monomer under denaturing condition, suggesting that dimerization of EIN3 was formed through non-covalent interactions.

EIN3 has been reported to recognize EBS and hence binds DNA in a sequence-specific manner [24]. To study how EIN3 recognizes its DNA targets, we first performed dynamic light scattering (DLS) analysis of EIN3. Our results showed that the hydrodynamic radius (Rh) of EIN3 82–352 was ~4.40 nm, which appeared to be of similar size of a 26–27 bp B-form duplex DNA (S5A Fig). Therefore, we designed a series of 28 bp probes to investigate the effect of specific sequences and their relative orientation within DNA to EIN3 binding. As shown in Fig 2B, EIN3 bound readily to *2EBS-S10* and *2EBS-S10-2*, which contain two known EBSs (ATGTAT and ATGTAC, respectively) [24, 30]. However, when we “flipped” the two EBSs, DNA binding was significantly reduced: for instance, binding of EIN3 to *2EBS-S10-R* was around 11-fold weaker than *2EBS-S10* [dissociation constants (*K*_d_): ~1.24 μM for *2EBS-S10-R* and ~0.11 μM for *2EBS-S10*] (Fig 2C and 2D). In addition, removing one of the two inverted EBSs from Probe *2EBS-S10* again significantly decreased binding affinity (*K*_d_ of ~0.69 μM for *1EBS-S10*), while removing both EBSs further decreased DNA-binding ability (*K*_d_ of ~2.28 μM for *0EBS-S10*) (Fig 2C and 2D). This was in contrast to mutations in flanking sequence of two EBSs, where no apparent effect on DNA binding was observed (S5B Fig). Taken together, these results suggest that EIN3 homodimer specifically recognizes two inverted EBSs within target DNA sequence, and the orientation of such EBSs is also important for optimal binding.

### Spacing between EBSs impacts binding affinity of EIN3

**Fig 3:**
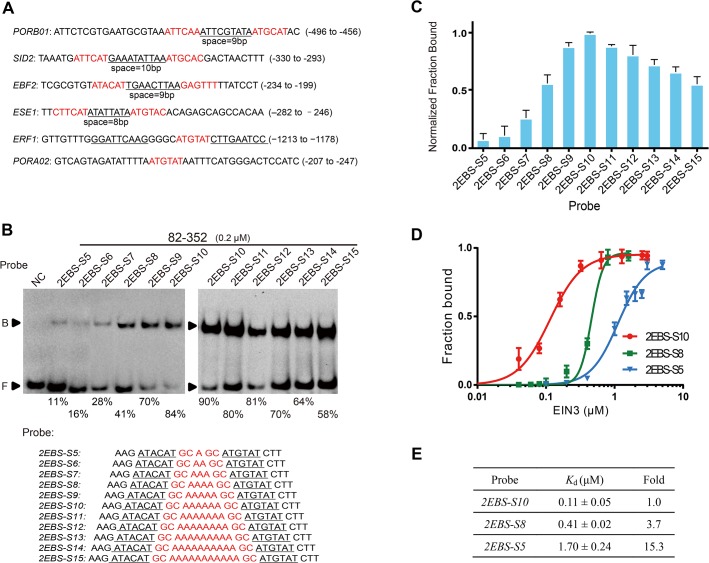
Spacing between EBSs constrains specific EIN3 binding. (A) Reported natural promoters of EIN3 targets. EBSs are shown in red. The spacing sequences between two EBSs are underlined and marked with the spacing lengths. All listed EIN3 targets have been proved by EMSA. (B) DNA-binding of EIN3 to probes with varying length of the spacing between EBSs. The sequences of the probes are listed in the lower panel. NC represents “negative control”, which is the free probe of *2EBS-S10* without the addition of any protein. (C) Relative binding data for 0.2 μM EIN3 82–352 to the probes in (B). The data were normalized to the fraction bound of *2EBS-S10*; error bars represented the standard deviation from three independent experiments. (D) Quantified binding data and (E) dissociation constants for probe *2EBS-S5*, *2EBS-S8*, and *2EBS-S10*. *K*_d_ values were obtained from three independent experiments.

The presence of two inverted EBSs have been found for many *in vivo* targets of EIN3 (*PORB01*, *SID2*, *EBF2*, *ESE1*, and etc) [30, 32, 34, 53] (Fig 3A), and such organization of EBSs presumably allows optimal binding of EIN3, also in agreement with our biochemical results shown above. Despite the fact that many EIN3 targets contain two inverted EBSs, a closer examination of such natural promoters reveals that the spacing between the two EBSs differ from each other. For instance, *ESE1* has an 8 bp spacing between two EBSs while *SID2* has a 10 bp spacing (Fig 3A). To investigate the effects of different spacing on EIN3 binding, we performed EMSA experiments using probes that have two invariant inverted ATGTAT EBSs but contain 5–15 bp spacing in between. As shown in Fig 3B and 3C, it was apparent that the spacing between the two EBSs could significantly affect the DNA-binding ability of EIN3: strongest binding showed up when the two EBSs were separated by a 10 bp spacing and gradually decreased when the spacing was either shortened from 10 bp to 5 bp or extended from 10 bp to 15 bp (Fig 3B and 3C; S6 Fig). In addition, the binding constants could vary up to 15 fold between *2EBS-S10* and *2EBS-S5* under the same experimental conditions, which was even greater than the difference between EIN3 binding to two and one EBS-containing sequences (*2EBS-S10* and *1EBS-S10*) and comparable to the difference between EIN3 binding to two and none EBS-containing sequences (*2EBS-S10* and *0EBS-S10*) (Fig 3D and 3E). Even the *K*_d_ of *2EBS-S8* was 3-fold higher than *2EBS-S10*, further indicating the significant impact of spacing on DNA binding. To rule out the potential roles of the spacing sequence, we made mutations in the spacing sequences and found that EIN3 binding was not affected (S6A and S6B Fig). Moreover, we also showed that the effect of spacing on the preferential binding of EIN3 was independent on the specific EIN3 truncations used in our study: similar results were also obtained on EIN3 91–352, 91–306 and 174–306, respectively (S6 Fig). Hence, our results reveal that the length of spacing between EBSs can significantly constrain EIN3’s affinity towards cognate DNA target and that a 10 bp spacing is optimal for EIN3 binding.

### Palindromic repeat sequence is not necessary for EIN3 binding

**Fig 4:**
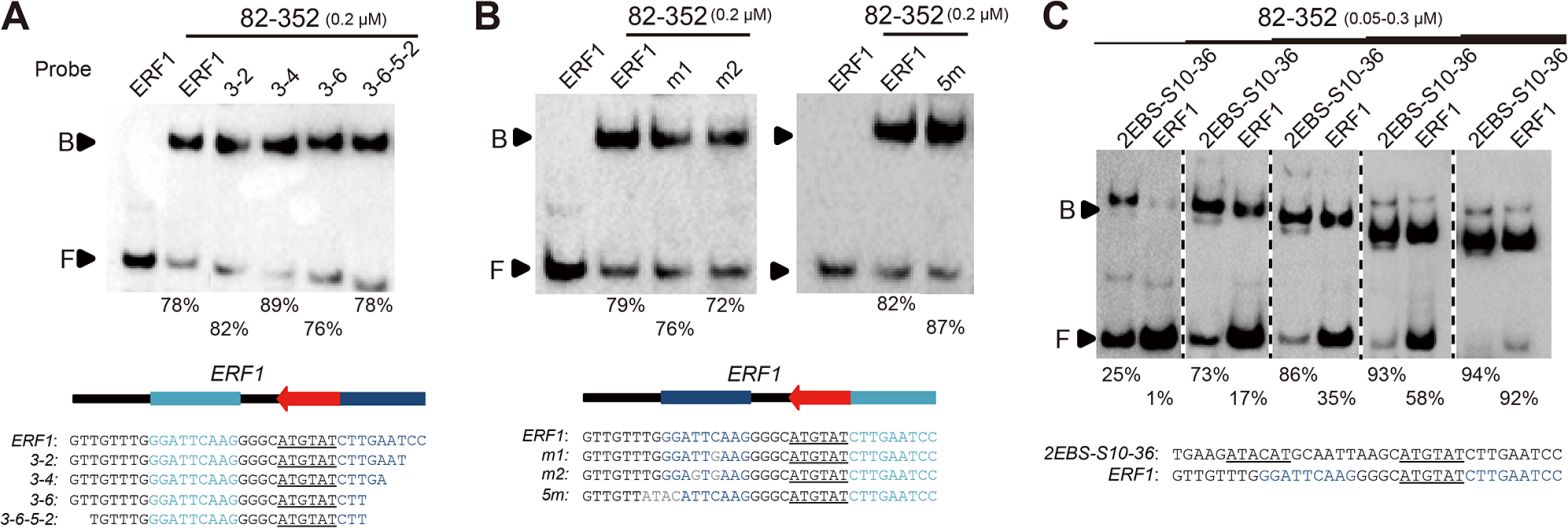
Palindromic repeats in *ERF1* promoter are not necessary for EIN3 binding. DNA-binding ability of EIN3 82–352 to (A) *ERF1* probes with 3’ palindromic repeat gradually deleted and (B) *ERF1* probes with 5’ palindromic repeat mutated. In schematic structural diagram of *ERF1*, (dark) blue filled boxes and red arrow indicate palindromic repeats and EBS, respectively. (C) Comparisons of binding affinity of 36 bp probe *2EBS-S10-36* and *ERF1*, demonstrating that EIN3 bound tighter to the two EBS-containing *2EBS-S10-36* than *ERF1*.

Previous studies reported that palindromic repeats could also be important for DNA binding of EIN3 [24]. For example, one of the prominent EIN3 target, *ERF1* contains two palindromic repeats flanking one central EBS; although *in vivo* targets of EIN3 can have either one or two EBS, such nearby palindromic repeats appears to be unique for *ERF1*. To determine whether the palindromic repeats are necessary for DNA binding, we systematically deleted/mutated the palindromic sequences from a 36 bp fragment of *ERF1* promoter, which was widely used in previous studies [24, 28]. Deletions of the 3’ palindromic repeat in *ERF1* (resulting in a shorter probe) did not affect binding ability (Fig 4A); additionally, mutations to the 5’ palindromic repeat in the DNA fragment did not affect binding ability either (Fig 4B). Moreover, binding affinity of EIN3 to the 36 bp *ERF1* was significantly lower than the 36 bp *2EBS-S10-36* probe (which contains two inverted EBS repeats) (Fig 4C). As a result, we conclude that palindromic repeats in *ERF1* promoter are not necessary for EIN3 binding; it is the EBS that is important for the interaction with EIN3.

To further validate the correlation between EIN3 DNA-binding affinity and EIN3 transcriptional activity, we performed transient transcriptional activity assays using *Arabidopsis* protoplast (S7 Fig). Three 36 bp probes (*1EBS-S10-36*, *2EBS-S10-36* and *ERF1*) were selected to drive *LUC* gene expression (S7A Fig). We monitored the LUC/REN ratio, which reflects *in vivo* EIN3 transcriptional activity upon EIN3 co-expression. Consistent with the DNA-binding results from our biochemical experiments (Figs 2B and 4C), the two EBS-containing plasmid displayed higher LUC/REN ratio than the two one EBS-containing plasmid (S7B Fig). Hence, higher affinity of EIN3 to DNA also demonstrates higher transcriptional activation.

### Crystal structure of the core DNA-binding domain of EIN3

**Fig 5:**
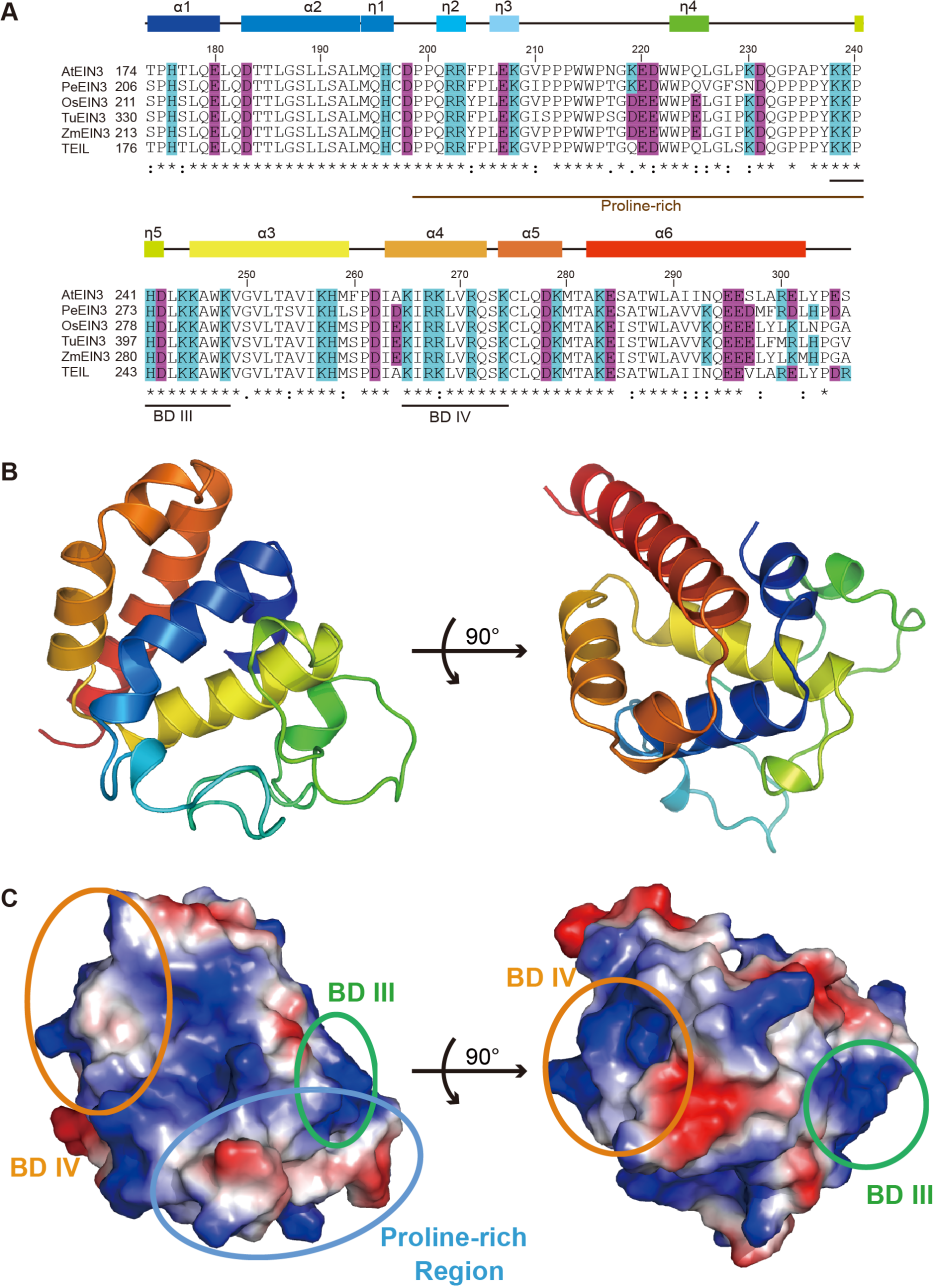
Crystal structure of EIN3 core DNA-binding domain (PDB code 4ZDS). (A) Sequence alignment of the core DBD of EIN3 proteins in different species produced by the Clustal Omega program. Sequences of the EIN3 proteins from *Arabidopsis thaliana* (AtEIN3, O24606), *Phalaenopsis equestris* (PeEIN3, Q711J2), *Oryza sativa* (OsEIN3, Q8W3M0), *Triticum urartu* (TuEIN3, M8A6N5), *Zea mays* (ZmEIN3, B6U809), and *Nicotiana tabacum* (TEIL, Q9ZWK1) are obtained from the UniProt database. Numbers above the sequences are for AtEIN3. Basic (His, Arg and Lys) and acidic (Asp and Glu) residues presented here are colored blue and purple, respectively. Colored boxes above the sequence alignment indicate helical structures of EIN3 core DBD. Identical and similar residues are marked by * and: respectively. BD III, BD IV and proline-rich region defined in Fig 1B are also indicated, and the sequence alignment includes the second highly conserved region in Fig 1B. (B) Crystal structure of EIN3 core DBD in two orientations (90° rotation along the indicated axis), which has the same coloring with the sequence alignment in (A). (C) Electrostatic surface potentials of EIN3 core DBD in the same orientation to (B). The proline-rich region, BD III and BD IV are indicated by blue, green and orange circles, respectively.

The core DBD (174–306) of EIN3 we identified contains the entire continuous conserved amino acid residues within the EIN3/EIL1 family (Fig 5A), and also contains BD III, BD IV and the proline-rich region. Moreover, the core DBD is capable of binding to various EIN3 DNA targets, albeit with lower affinity comparing to the optimal DBD (Fig 1E and S6H Fig). Furthermore, although the solution structure of EIL3 major DBD is solved [38], whether or not EIL3 can bind to EBS-containing DNAs is still under debate [24, 38]; more importantly, the physiological function of EIL3 in Arabidopsis is distinct from other EIL family members [39]. Thus, we purified and crystallized the core DBD to gain mechanistic insights of EIN3 at atomic resolution.

Firstly, we used the solution structure of EIL3 (PDB code 1WIJ) as the model for molecular replacement, however no solutions could be found. We then solved the structure of the core DBD using single-wavelength anomalous diffraction (SAD) on a seleno-methionine derivative; the resulting model was used for molecular replacement on the native structure, which was solved to 1.78 Å (R_work_: 17.4% and R_free_: 21.8%) (Table 1) (PDB code 4ZDS). The structure revealed that the EIN3 core DBD exists as a monomer and is composed of six α-helices (α1-α6) and five short helical turns (η1-η5) (Fig 5A and 5B). In fact, the core DBD was also a monomer in solution in the absence of DNA (S8 Fig), which is in agreement with the previous NMR study [38]. Presumably, this is due to the disruption of N-terminal amino acid residues that are important for dimerization. The core DBD structure also contains several amino acid residues (Lys245, Pro216 and Thr174) that are critical for EIN3 function (S9 Fig). Structure similarity searches using the DALI server [54] showed that EIL3 is the only protein that shares structural homology to the DBD of EIN3, with no other homologs found in the Protein Data Bank. The N-terminal region (1–340) of EIL3 is quite conserved compared to EIN3 1–352 (~52.8% sequence identity), and the overall structure of EIN3 core DBD has a root-mean-square deviation (RMSD) of 3.32 Å with the solution structure of EIL3 [38] (S10 Fig). Therefore, together with previous observations, we conclude that the structures of EIN3 and EIL3 represent a novel fold of DBD.

## Discussion

EIN3/EIL1 proteins act as master regulators in plant signaling network, recognizing and binding to specific promoters (EBS-containing promoters) and thus modulating target genes expression. To date, numerous targets of EIN3 have been identified and majority of them are regulated by ethylene [26]. However, the molecular mechanism of the interactions between EIN3 and its targets is still unclear. Using systematically varied recombinant proteins, we identify 82–352 fragment and 174–306 fragment as the optimal and core DBD of EIN3, respectively. We show that EIN3 homodimer binds to DNA probes containing two inverted EBSs; the EBS sequences are critical to allow specific and optimal binding of EIN3. Notably, our results reveal that the length of spacing between two EBSs can clearly impact binding affinity of EIN3, while the sequence of the spacing does not affect binding. And 10 bp spacing is optimal for EIN3 binding. In addition, palindromic repeats appear to be dispensable for DNA binding of EIN3, as demonstrated in the case of *ERF1*, one well-known target of EIN3. Furthermore, we solved a high resolution structure of the core DBD of EIN3, which provides the structural basis for the mechanistic views of several key amino acid residues involved in the biological function of EIN3.

Our systematic comparisons revealed that DNA-binding affinity decreased gradually from EIN3 1–352 to 35–352 and 46–352, whereas truncations 68–352, 82–352, 91–352, and 98–352 exhibited significantly increased DNA-binding ability (Fig 1C and 1D). Such observations hint that the DNA-binding activity of EIN3 could be autoinhibited by its N-terminal sequence (~1–50). In fact, autoinhibition of DNA-binding capacity has been observed for several other transcription factors: for instance, E26 transformation-specific transcription factor (Ets-1) is autoinhibited by a conformationally disordered serine-rich region (SRR), and phosphorylation of serines within the SRR has been shown to enhance such autoinhibition [55, 56]. In the case of EIN3, four Ser and one Thr residues are present within the N-terminal sequence (1–50), implying the possibility of being post-translationally modified and utilized for regulation. In addition to putative “self-regulation”, DNA binding of EIN3 can also be regulated through interacting with other proteins. For instance, we reported in a previous study that MYC2 interacts with EIN3 and interferes with its DNA binding and transcriptional activities [29]. In this study, we showed that EIN3 can form a homodimer in solution and such dimerization, which is independent of DNA binding events, appears to enhance DNA binding since dimerized EIN3 truncations (e.g. 82–352) has better affinity than monomeric EIN3 truncations (e.g. 174–306). Thus, one possibility is that MYC2 could interfere with the formation of EIN3 homodimer and hence interfere with DNA-binding ability of EIN3.

Previous studies, together with ours, have shown that EBS sequences are essential for DNA binding of EIN3 [24, 37]. As a matter of fact, among the many known targets of EIN3, both one and two EBS-containing targets have been reported (for example, *SID2* and *ESE1* have two inverted EBSs, while *ERF1* and *PORA02* have only one EBS) [24, 30, 32, 34] (Fig 3A). Therefore, it will be interesting to see whether or not different target genes can be preferentially recognized and bound by EIN3 based on the number of EBS present in the promoter region, particularly given the fact that the cellular concentration of EIN3 proteins are constantly subjected to regulation (e.g. by EBF1/2) [18, 19]. Results from our EMSA experiments revealed that EIN3 bound to DNA probes bearing two EBSs 6-fold tighter than probes with one EBS (Fig 2C and 2D). Moreover, we found that the length, but not the sequence, of the spacing between two inverted EBSs was able to impact the binding affinity of EIN3 (Fig 3). As natural targets of EIN3 have varied length of spacing between two EBSs, such spacing length (and perhaps also the spatial phase between two adjacent EBS) may add one additional layer of complexity to the transcriptional regulation controlled by EIN3. Furthermore, an optimized probe sequence was identified and could be applied to better reflect the transcriptional activity of EIN3, i.e. the tandem copies of *2EBS-S10-36* could be fused with luciferase or GUS genes and be used in transient assay or stable transgenic lines, defined as enhanced EBS reporter system and replacing the previous 5×EBS::GUS version [27, 28]. In addition, our analyses on EIN3 target sequence and optimal DBD could also help to obtain EIN3/dsDNA complex structures in future.

Our work provides the crystal structure of EIN3 core DBD and reveals the structural basis of key domains and amino acid residues for EIN3 function. The core DBD 174–306 contains two essential basic domains for DNA binding: BD III (K238-K248) and BD IV (K265-K274). BD III and BD IV are highly positively charged, and are located on the surface of the truncation, but nearly on opposing sides of the truncation (Fig 5C). Therefore, it seems unlikely that both basic domains are simultaneously involved in direct DNA binding. Several amino acids that are critical for EIN3 function have been identified previously: for instance, a single mutation K245N shows a loss of ethylene-mediated effects [25]; P216S mutation abolishes regulation of the ethylene induced triple response, while remains fully functional in repressing plant disease resistance [32]; and T174 of EIN3 can be phosphorylated by the MKK9–MPK3/6 modules, although the role of such post-translational modification in ethylene signaling appears to be under debate [21]. These amino acid residues are all present in our crystal structure: residue 245 belongs to α3 in the core DBD and falls within BD III, and the side chain of K245 forms two hydrogen bonds with the main chain carbonyl oxygen atoms of Q179 (α1) and L181 (between α1 and α2), respectively (S9A Fig). Proline 216 resides in the proline-rich region of EIN3 and is involved in a hydrophobic interaction network of the loop region (S9B Fig and S11 Fig). When Pro is replaced by the hydrophilic Ser residue, such interaction may be weakened. Interestingly, binding ability of EIN3 to the ERF1 promoter was not affected by the Pro-to-Ser mutation (S9C Fig). T174 belongs to α1 and its hydroxyl group forms a hydrogen bond to the side chain of E295 (α6) (S9D Fig). However, an EIN3/dsDNA o complex structure would be desired to unambiguously establish exact molecular interactions of these key amino acids.

An important question in plant signaling network is how EIN3/EIL1 regulate such a wide range of target genes. In this work, we studied in details the DNA-binding properties of EIN3 and revealed the mechanistic insights into the interplay between EIN3 and different DNA targets. These results, as well as the EIN3 structure presented here, will open new opportunities to the sequence-dependent gene regulation in plant signaling network.

## Supporting Information

S1 Fig Probe purification. (A) The HPLC chromatograms of *2EBS-S10-R* (reverse strand of *2EBS-S10)*, *2EBS-S10-F* (forward strand of *2EBS-S10)* and annealed *2EBS-S10* (forward strand: reverse strand = 1:2) are shown in the upper, middle and lower panel, respectively. DNA absorbance was monitored at 260 nm, as DNA was eluted from the column using a NaCl gradient of 0.4–1.0M in 10 mM Tris–HCl (pH 7.5) buffer over the course of 12.5 min. As the NaCl gradient was applied, *2EBS-S10-R* was first eluted (~3.1 min), followed by *2EBS-S10-F* (~3.4 min) and finally the desired *2EBS-S10* dsDNA probe (~4.9 min). (B) 16% polyacrylamide 8 M urea denaturing gel and 8% polyacrylamide native gel analysis of peak collected in (A) as indicated after buffer exchange. (C) The *K*_d_ for EIN3 82–352 binding to purified double-stranded probes. Results were obtained from three independent replicates. (D) The HPLC chromatograms of annealed *2EBS-S10* (forward strand: reverse strand = 1:1).The content of annealed dsDNA probe was ~95%. (E) EMSA results of EIN3 82–352 with a 3’–biotin labeled *2EBS-S10-F* single-stranded probe.
(TIF)

S2 Fig Secondary structure prediction of EIN3 using the PSIPRED server. (TIF)

S3 Fig EMSA results of different truncated EIN3 proteins binding to *ERF1*. (A) EMSA of truncated EIN3 proteins binding to 3’–biotin labeled *ERF1*. The fraction bound is shown in the lower panel. Truncated EIN3 proteins (B) 82–352, (C) 114–352, (D) 141–352 and (E) 91–306 retained the ability to bind *ERF1*. Schematic structural diagrams of each truncated EIN3 protein are shown in the upper panel. (F) Circular dichroism analysis of 82–352 and 174–306 revealed alpha-helix was the major secondary structure for DBDs of EIN3.
(TIF)

S4 Fig Static light scattering and size exclusion chromatography of EIN3 82–382. (A) Analysis of EIN3 82–352 by static light scattering under low salt buffer (100 mM NaCl). Measured molecular weight (Mw) of EIN3 82–352 was 63.61 kDa (± 0.14 kDa). (B) Analysis of EIN3 82–352 by static light scattering under high salt buffer (300 mM NaCl). Measured molecular weight (Mw) of EIN3 82–352 was 63.45 kDa (± 0.18 kDa). (C) Size exclusion chromatography results of 82–352 under different salt concentration on Superdex 75 PG size-exclusion column (GE Healthcare).
(TIF)

S5 Fig Analysis of protein size and flanking sequence of the two EBSs. (A) Dynamic light scattering (DLS) analysis of EIN3 82–352. The protein size of EIN3 measured by DLS fitted the length of ~26–27 bp B-form DNA. (B) EMSA of EIN3 82–352 binding to probes with different flanking sequences of two EBSs. The fraction bound is shown right under the gel image. The sequences of probes are listed in the lower panel.
(TIF)

S6 Fig Probe containing ~10 bp spacing between two EBSs has highest affinity to EIN3. (A) EIN3 DNA-binding ability to probes with different spacing sequences between EBSs. The sequences of probes are listed in the lower panel. (B) Relative binding data for 0.2 μM EIN3 to probes in (A) with different spacing sequences. Values were normalized to the fraction bound of the probe *2EBS-S10*. (C-H) EMSA results of EIN3 truncations with different probes. EIN3 82–352 bound to probe (C) *2EBS-S10* tighter than (D) *2EBS-S15*. EIN3 (E) 91–352 and (F) 91–306 shared the same spacing constraint. EIN3 (G) 91–352 and (H) 174–306 retained the ability to bind *2EBS-S10*.
(TIF)

S7 Fig The EBS affinity with EIN3 is positively associated with EIN3 transcriptional activity. (A) The schematic diagram shows the constructs used in the transient transcriptional activity assays of (B). (B) Transient transcriptional activity assays show that the 36 bp *1EBS-S10-36*, *2EBS-S10-36* and *ERF1* sequences are activated by EIN3, respectively. The three kinds of reporter plasmids were cotransformed with the indicated effector plasmid. The Relative Fluorescence LUC/REN represents the relative LUC/REN ratio normalized by the control group. Data are means (±SD) of three biological replicates.
(TIF)

S8 Fig Analysis of EIN3 174–306 by SEC-MALS. 174–306 existed primarily in the form of monomer in the absence of DNA (measured Mw = 17.35 kDa ± 1.915%; predicted Mw = 17.69 kDa).
(TIF)

S9 Fig Spatial locations of key amino acid residues in EIN3 (PDB code 4ZDS). (A) K245 interacts with Q179 and L181. Electrostatic surface potential is also shown. When K245 is mutated to Asn, the interactions of K245 with Q179 and L181 could be disrupted and thus the folding of EIN3 could be affected. Alternatively, K245 could be directly involved in interactions with DNA, and mutation of a positively charged lysine residue to a neutral and shorter asparagine could also interfere with DNA binding. (B) Hydrophobic interaction network around P216. If Pro is replaced by the hydrophilic Ser residue, the side chain of Ser could flip around and face towards the solvent-exposed surface, causing local conformational change to the proline-rich region. (C) DNA-binding ability of EIN3 82–352 and P216S mutation to ERF1. As the binding ability of EIN3 to the *ERF1* promoter was not affected by the Pro-to-Ser mutation, the nonfunctional behavior of EIN3^P216S^ in regulation of “triple response” is not a consequence of altered protein-DNA interactions of EIN3; instead, it is likely that conformational changes caused by the P216S mutation results in altered protein-protein interactions. In addition, Pro216 resides in a peptide sequence “PPPWWP” (211–216), conforming to the core ligand motif PxxP of SH3 domains (where x denotes any amino acid). Although no SH3 domain-containing proteins are currently known to interact with EIN3, it remains to be investigated whether such interacting-partners could exist and account for this distinct functional behavior of EIN3^P216S^. (D) Spatial locations of T174. Green dashed lines indicate hydrogen-bonds, while grey dashed lines indicate the distance between nitrogen atom and oxygen atom in side-chain. When T174 is phosphorylated, the hydrogen bond between T174 and E295 (α6) could be disfavored by the negative charge and steric clash of the large phosphate group. Interestingly, surrounding residue T174, there are positively charged K257 (α3) and also Q294 (α6); it is conceivable to speculate whether their side chains could interact with the negatively charged phosphate group, and hence further affect the local conformation of the protein upon phosphorylation.
(TIF)

S10 Fig Comparison of EIN3 core DBD with EIL3 major DBD (PDB code 1WIJ). (TIF)

S11 Fig Crystal structure of EIN3 core DBD. BD III and BD IV are in dark blue; “PPPWWP” (211–216) is in orange (P216 is marked by red).
(TIF)

S1 File Liquid chromatography-tandem mass spectrometry analysis of EIN3 82–352. (PDF)

S1 Table Oligo sequences used in experiments. (XLSX)
