# Supplementary figures and images for: Biochemical and Structural Insights into the Mechanism of DNA Recognition by Arabidopsis ETHYLENE INSENSITIVE3
Source: PLoS One. 2015 Sep 9;10(9):e0137439. doi: 10.1371/journal.pone.0137439 (PMC4564277; doi:10.1371/journal.pone.0137439)

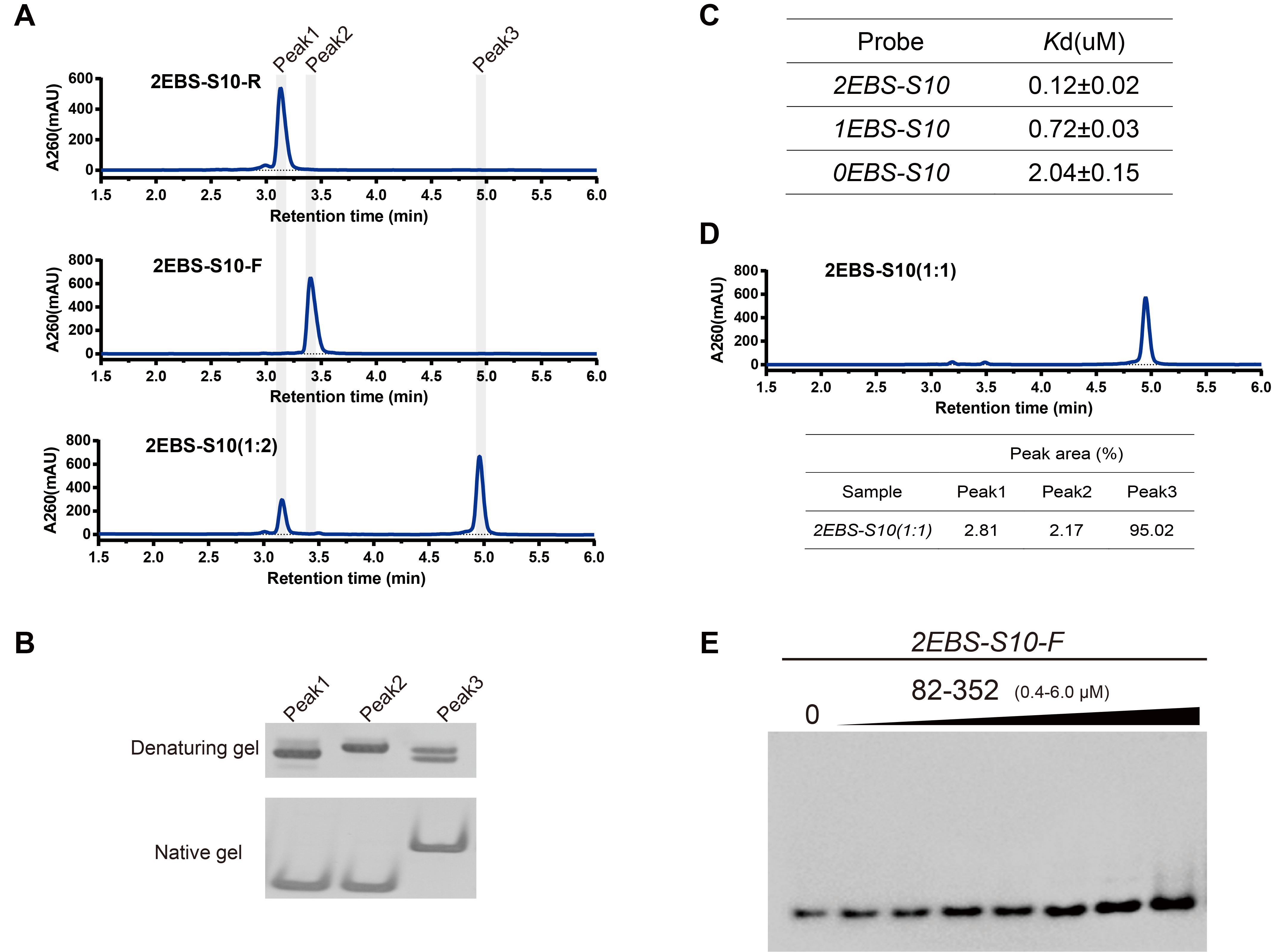

Supplement: S1 Fig — (A) The HPLC chromatograms of 2EBS-S10-R (reverse strand of 2EBS-S10), 2EBS-S10-F (forward strand of 2EBS-S10) and annealed 2EBS-S10 (forward strand: reverse strand = 1:2) are shown in the upper, middle and lower panel, respectively. DNA absorbance was monitored at 260 nm, as DNA was eluted from the column using a NaCl gradient of 0.4–1.0M in 10 mM Tris–HCl (pH 7.5) buffer over the course of 12.5 min. As the NaCl gradient was applied, 2EBS-S10-R was first eluted (~3.1 min), followed by 2EBS-S10-F (~3.4 min) and finally the desired 2EBS-S10 dsDNA probe (~4.9 min). (B) 16% polyacrylamide 8 M urea denaturing gel and 8% polyacrylamide native gel analysis of peak collected in (A) as indicated after buffer exchange. (C) The K d for EIN3 82–352 binding to purified double-stranded probes. Results were obtained from three independent replicates. (D) The HPLC chromatograms of annealed 2EBS-S10 (forward strand: reverse strand = 1:1).The content of annealed dsDNA probe was ~95%. (E) EMSA results of EIN3 82–352 with a 3’–biotin labeled 2EBS-S10-F single-stranded probe. (TIF) [file pone.0137439.s001.tif]

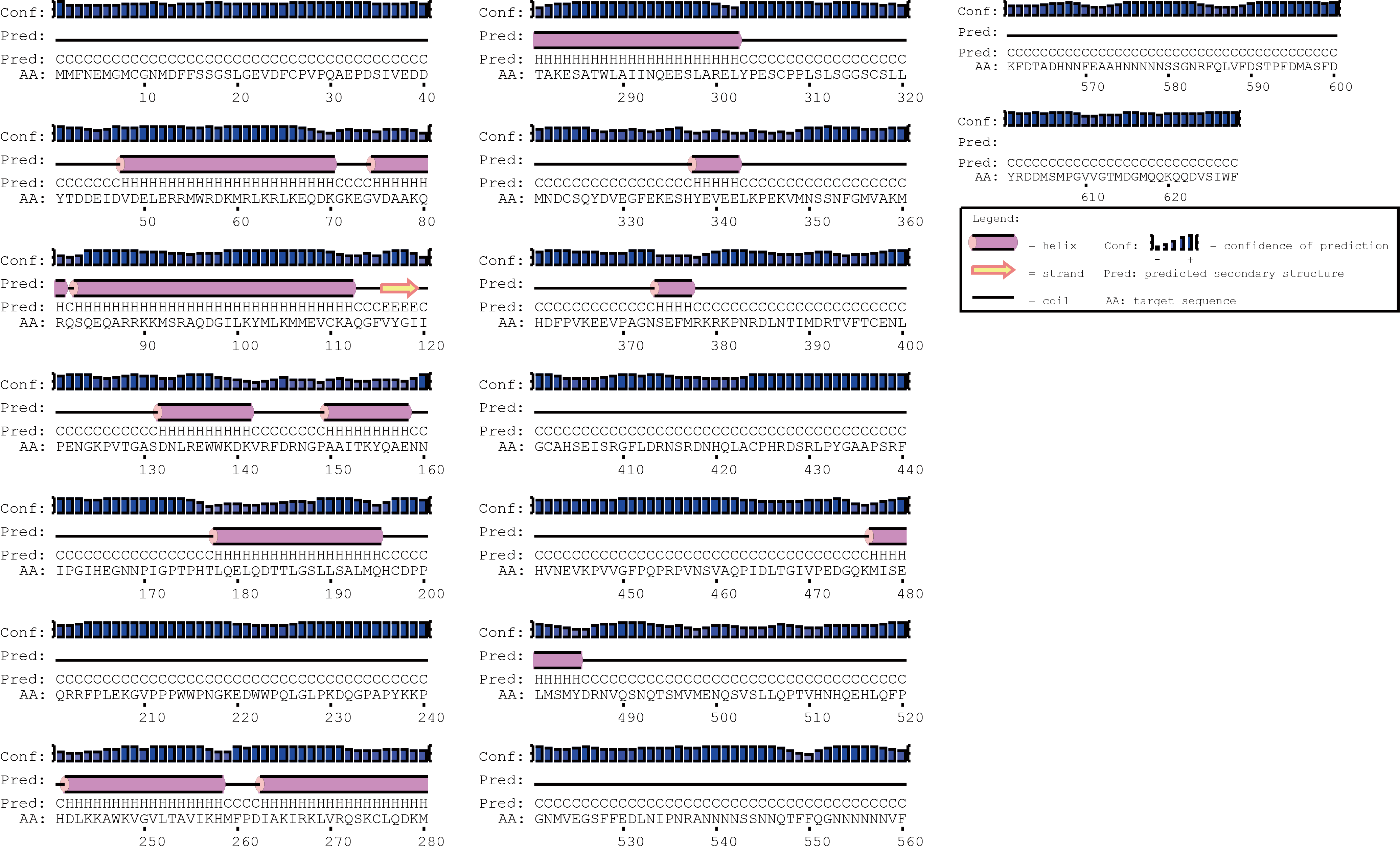

Supplement: S2 Fig — (TIF) [file pone.0137439.s002.tif]

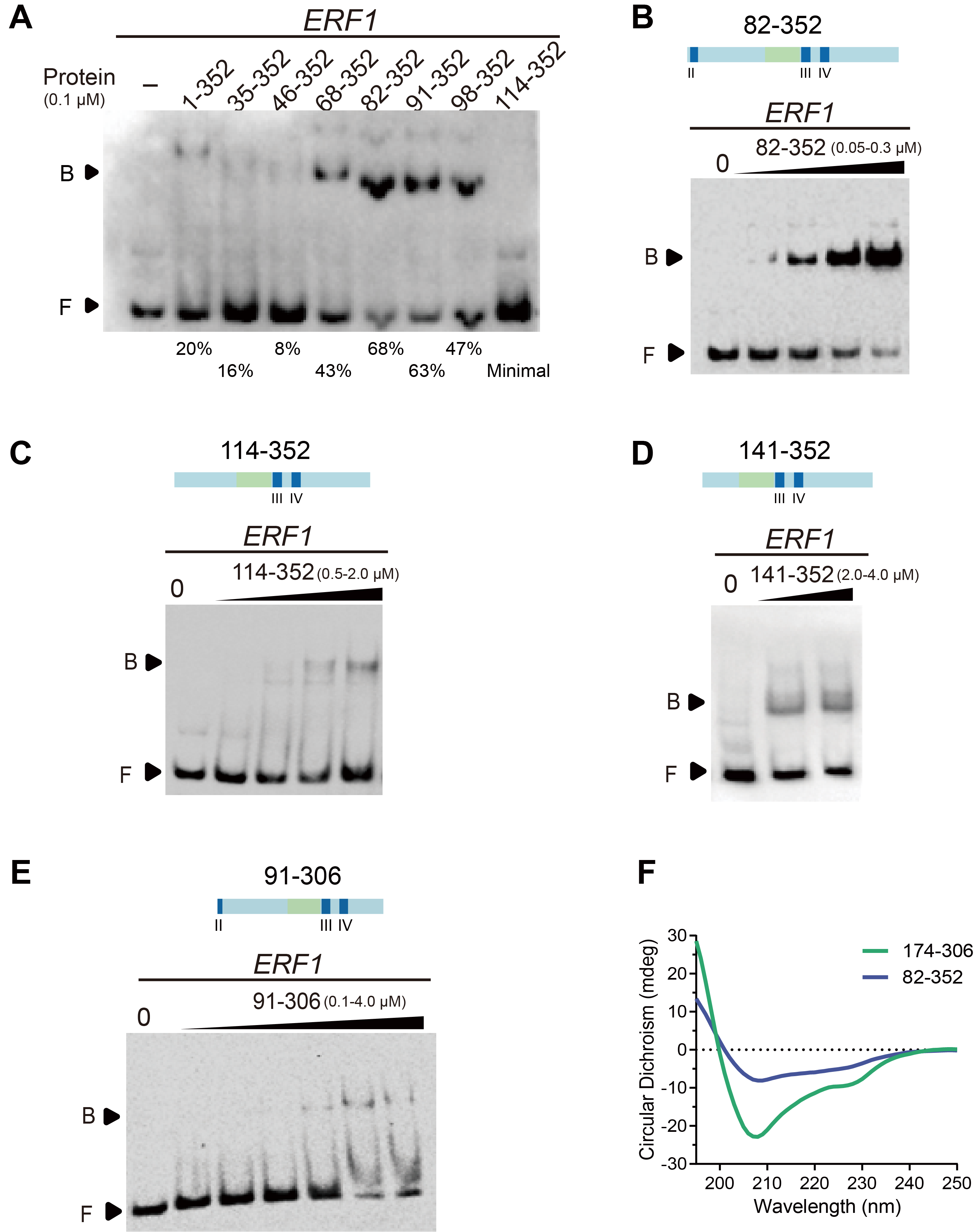

Supplement: S3 Fig — (A) EMSA of truncated EIN3 proteins binding to 3’–biotin labeled ERF1. The fraction bound is shown in the lower panel. Truncated EIN3 proteins (B) 82–352, (C) 114–352, (D) 141–352 and (E) 91–306 retained the ability to bind ERF1. Schematic structural diagrams of each truncated EIN3 protein are shown in the upper panel. (F) Circular dichroism analysis of 82–352 and 174–306 revealed alpha-helix was the major secondary structure for DBDs of EIN3. (TIF) [file pone.0137439.s003.tif]

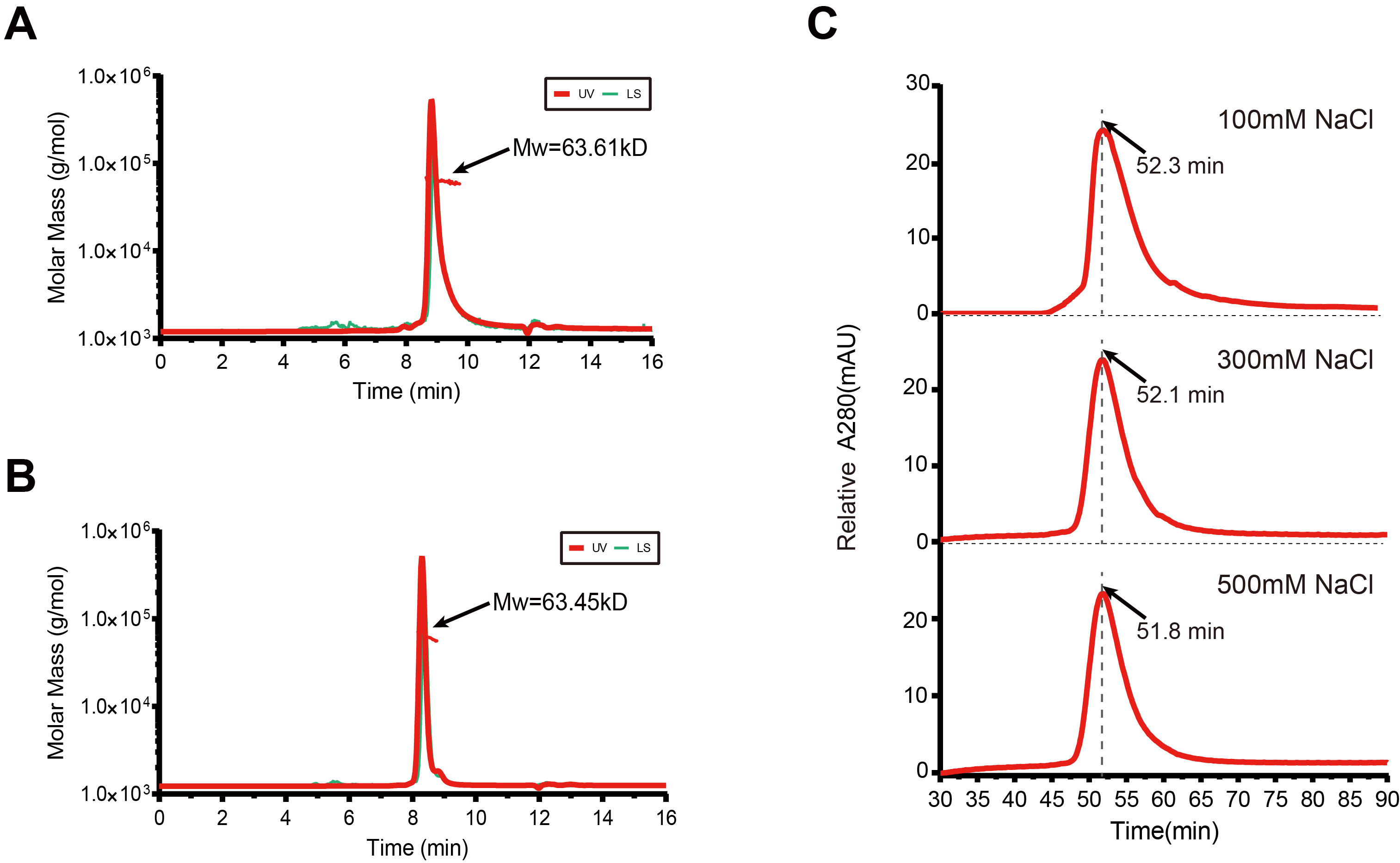

Supplement: S4 Fig — (A) Analysis of EIN3 82–352 by static light scattering under low salt buffer (100 mM NaCl). Measured molecular weight (Mw) of EIN3 82–352 was 63.61 kDa (± 0.14 kDa). (B) Analysis of EIN3 82–352 by static light scattering under high salt buffer (300 mM NaCl). Measured molecular weight (Mw) of EIN3 82–352 was 63.45 kDa (± 0.18 kDa). (C) Size exclusion chromatography results of 82–352 under different salt concentration on Superdex 75 PG size-exclusion column (GE Healthcare). (TIF) [file pone.0137439.s004.tif]

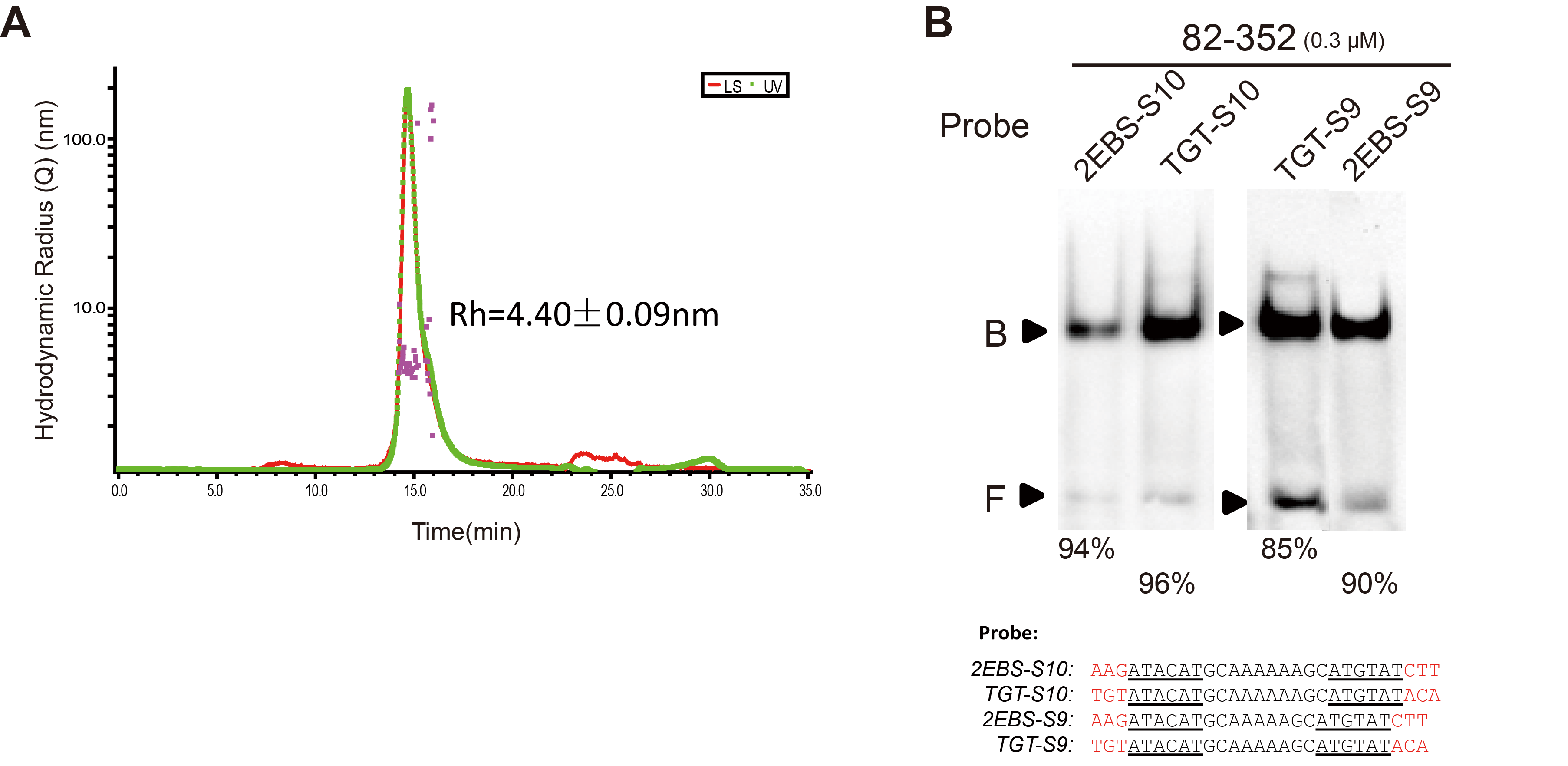

Supplement: S5 Fig — (A) Dynamic light scattering (DLS) analysis of EIN3 82–352. The protein size of EIN3 measured by DLS fitted the length of ~26–27 bp B-form DNA. (B) EMSA of EIN3 82–352 binding to probes with different flanking sequences of two EBSs. The fraction bound is shown right under the gel image. The sequences of probes are listed in the lower panel. (TIF) [file pone.0137439.s005.tif]

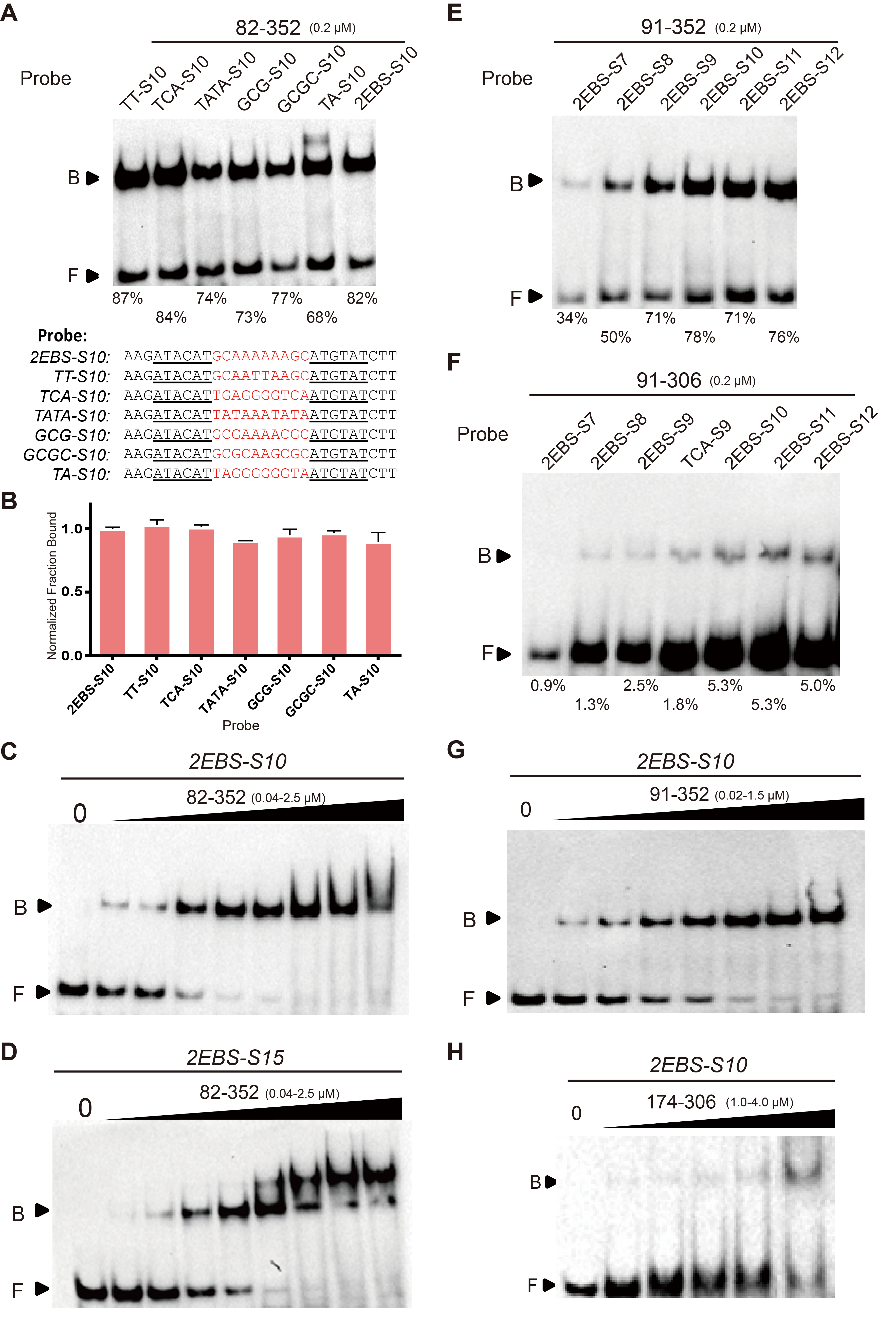

Supplement: S6 Fig — (A) EIN3 DNA-binding ability to probes with different spacing sequences between EBSs. The sequences of probes are listed in the lower panel. (B) Relative binding data for 0.2 μM EIN3 to probes in (A) with different spacing sequences. Values were normalized to the fraction bound of the probe 2EBS-S10. (C-H) EMSA results of EIN3 truncations with different probes. EIN3 82–352 bound to probe (C) 2EBS-S10 tighter than (D) 2EBS-S15. EIN3 (E) 91–352 and (F) 91–306 shared the same spacing constraint. EIN3 (G) 91–352 and (H) 174–306 retained the ability to bind 2EBS-S10. (TIF) [file pone.0137439.s006.tif]

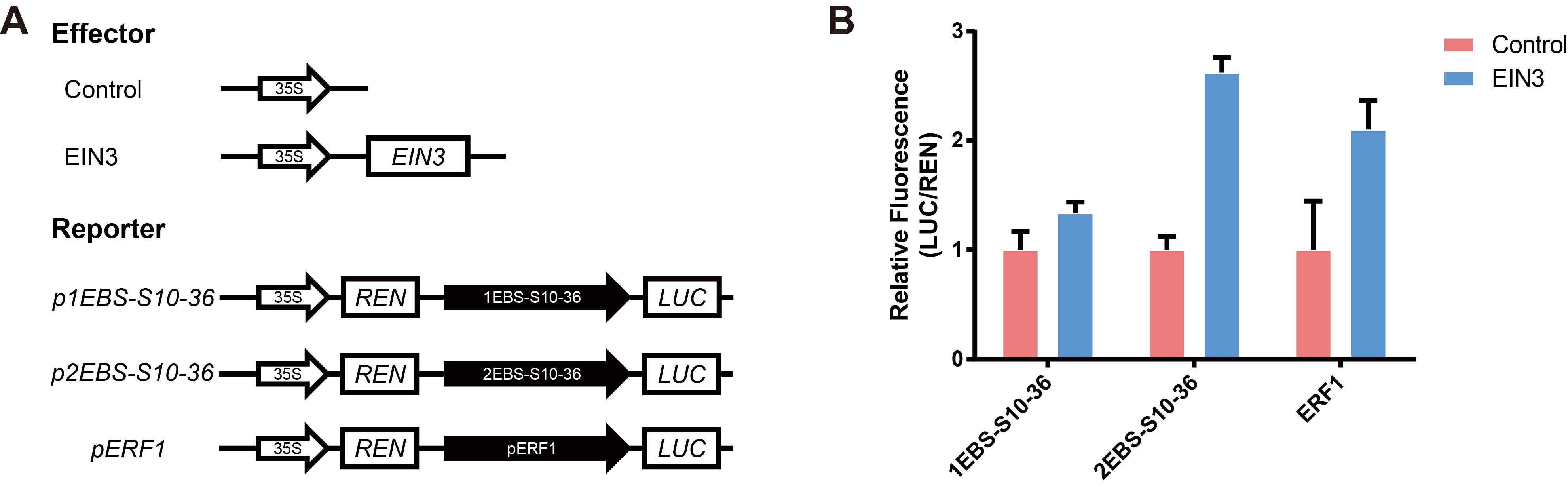

Supplement: S7 Fig — (A) The schematic diagram shows the constructs used in the transient transcriptional activity assays of (B). (B) Transient transcriptional activity assays show that the 36 bp 1EBS-S10-36, 2EBS-S10-36 and ERF1 sequences are activated by EIN3, respectively. The three kinds of reporter plasmids were cotransformed with the indicated effector plasmid. The Relative Fluorescence LUC/REN represents the relative LUC/REN ratio normalized by the control group. Data are means (±SD) of three biological replicates. (TIF) [file pone.0137439.s007.tif]

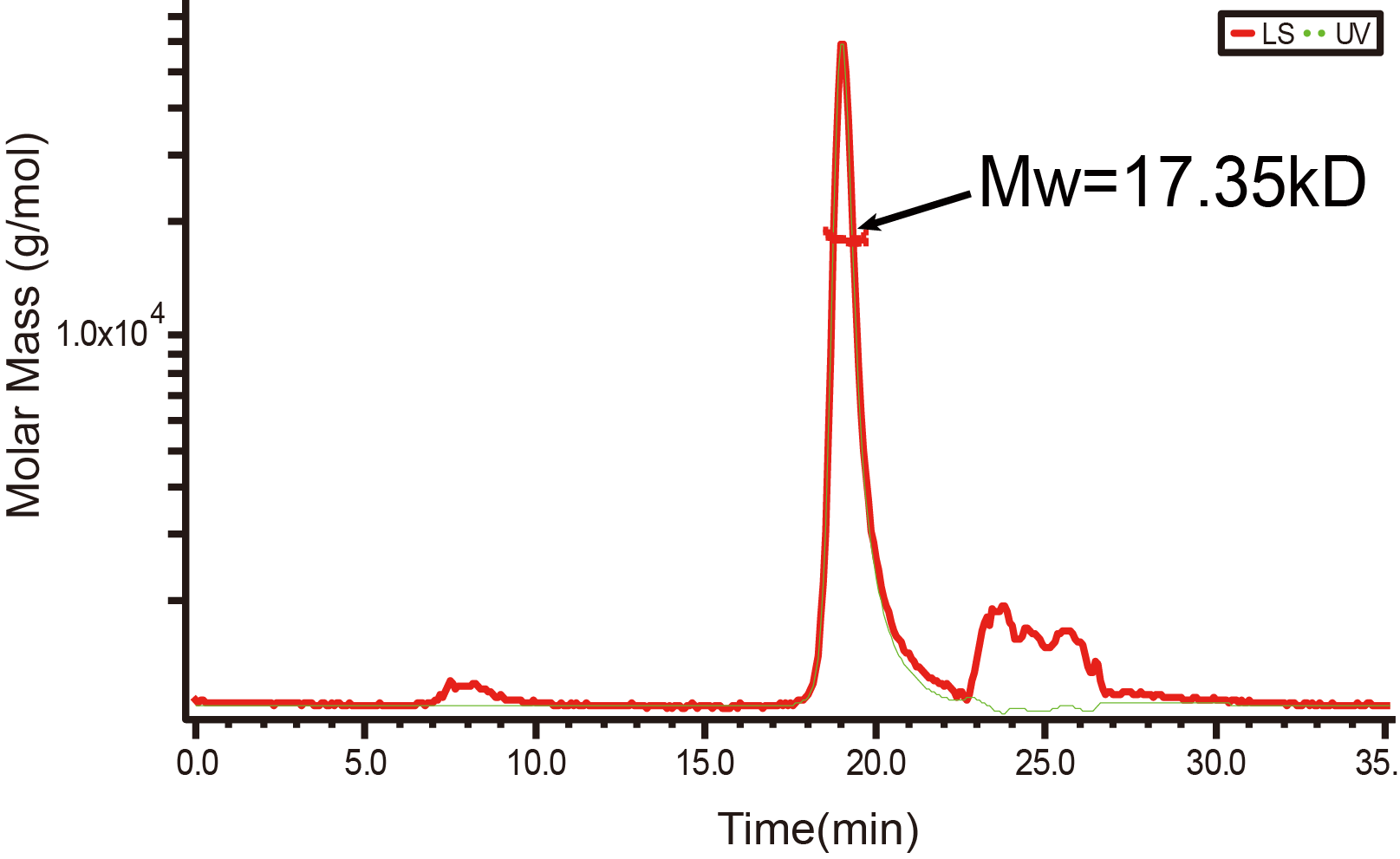

Supplement: S8 Fig — 174–306 existed primarily in the form of monomer in the absence of DNA (measured Mw = 17.35 kDa ± 1.915%; predicted Mw = 17.69 kDa). (TIF) [file pone.0137439.s008.tif]

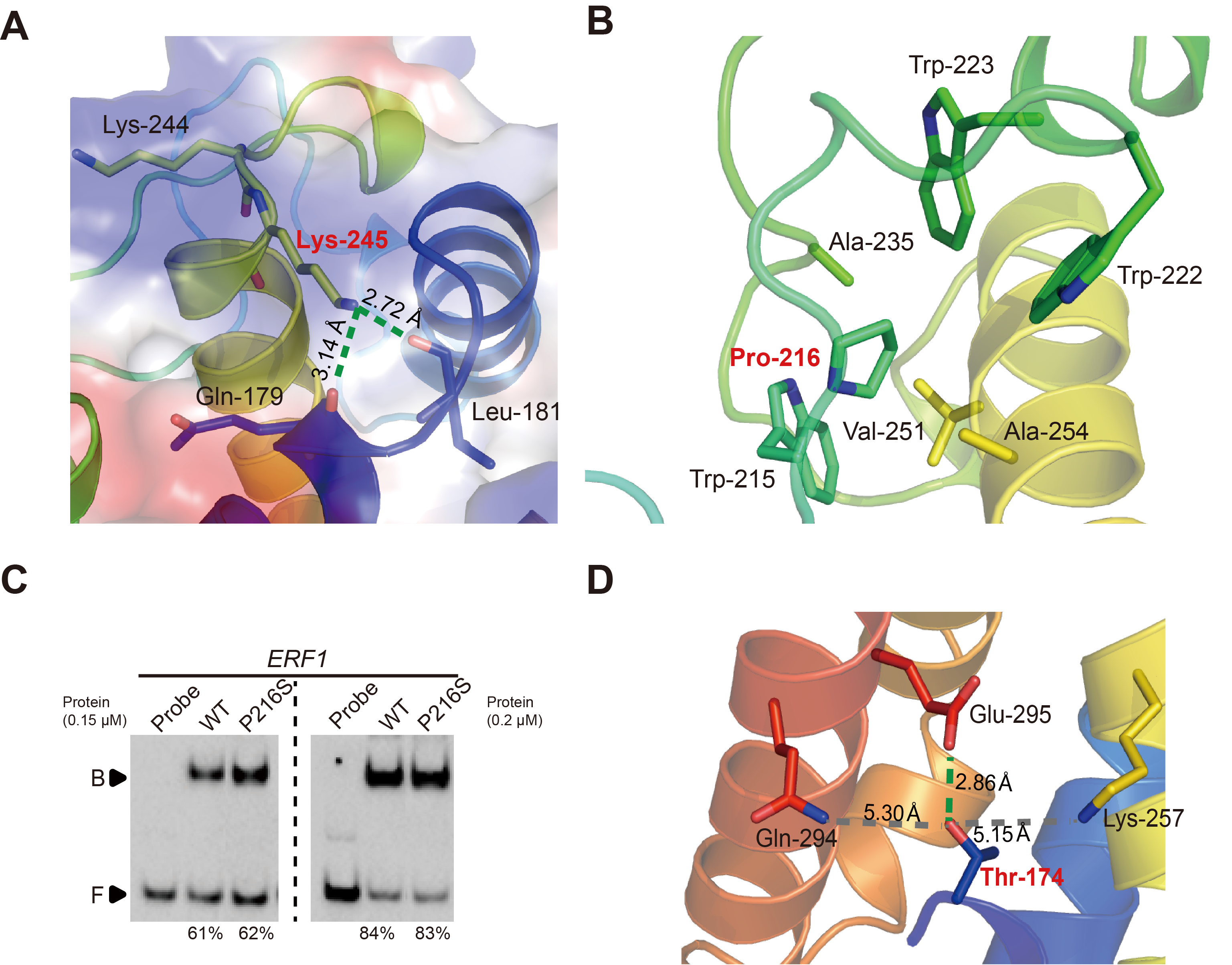

Supplement: S9 Fig — (A) K245 interacts with Q179 and L181. Electrostatic surface potential is also shown. When K245 is mutated to Asn, the interactions of K245 with Q179 and L181 could be disrupted and thus the folding of EIN3 could be affected. Alternatively, K245 could be directly involved in interactions with DNA, and mutation of a positively charged lysine residue to a neutral and shorter asparagine could also interfere with DNA binding. (B) Hydrophobic interaction network around P216. If Pro is replaced by the hydrophilic Ser residue, the side chain of Ser could flip around and face towards the solvent-exposed surface, causing local conformational change to the proline-rich region. (C) DNA-binding ability of EIN3 82–352 and P216S mutation to ERF1. As the binding ability of EIN3 to the ERF1 promoter was not affected by the Pro-to-Ser mutation, the nonfunctional behavior of EIN3P216S in regulation of “triple response” is not a consequence of altered protein-DNA interactions of EIN3; instead, it is likely that conformational changes caused by the P216S mutation results in altered protein-protein interactions. In addition, Pro216 resides in a peptide sequence “PPPWWP” (211–216), conforming to the core ligand motif PxxP of SH3 domains (where x denotes any amino acid). Although no SH3 domain-containing proteins are currently known to interact with EIN3, it remains to be investigated whether such interacting-partners could exist and account for this distinct functional behavior of EIN3P216S. (D) Spatial locations of T174. Green dashed lines indicate hydrogen-bonds, while grey dashed lines indicate the distance between nitrogen atom and oxygen atom in side-chain. When T174 is phosphorylated, the hydrogen bond between T174 and E295 (α6) could be disfavored by the negative charge and steric clash of the large phosphate group. Interestingly, surrounding residue T174, there are positively charged K257 (α3) and also Q294 (α6); it is conceivable to speculate whether their side chains could inte [file pone.0137439.s009.tif]

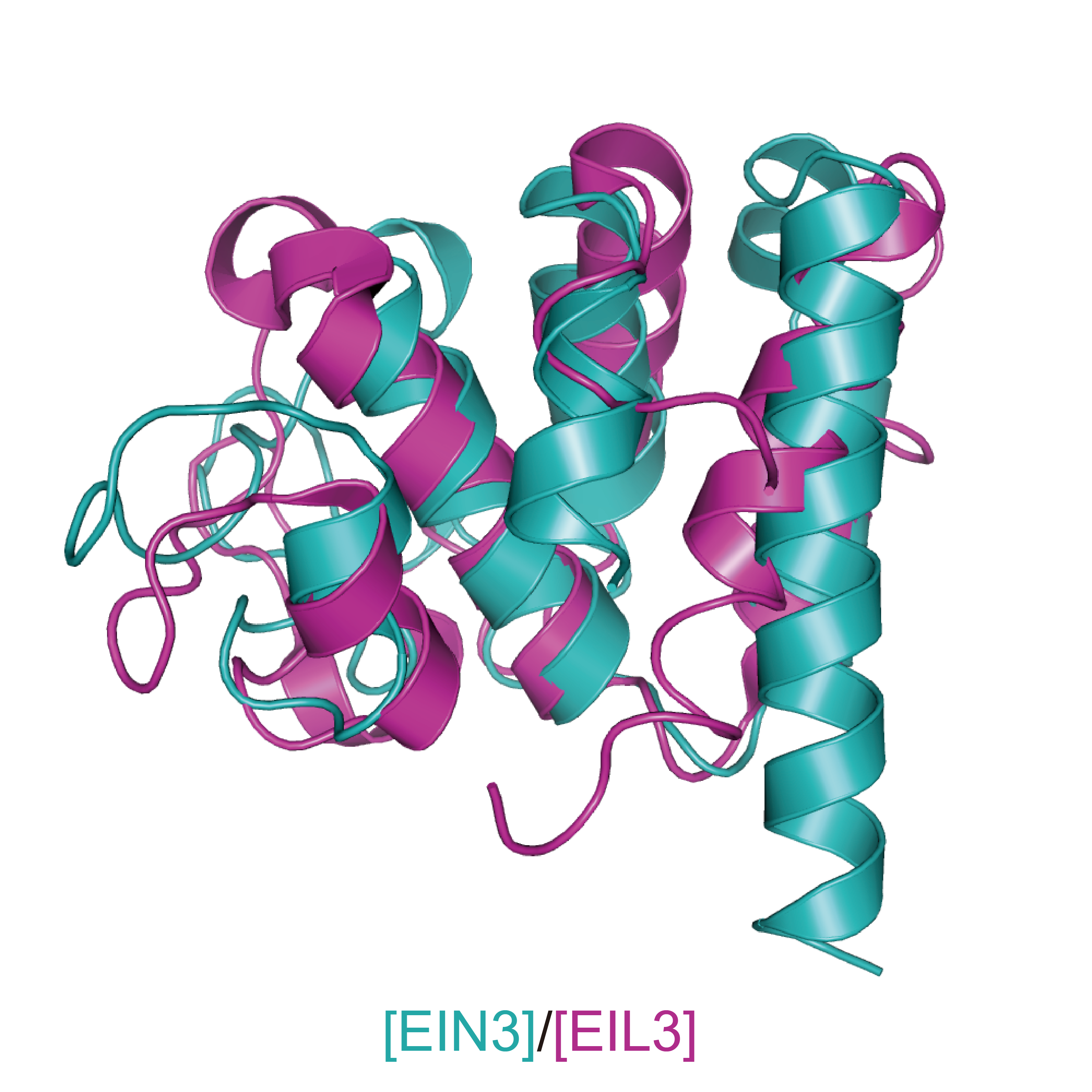

Supplement: S10 Fig — (TIF) [file pone.0137439.s010.tif]

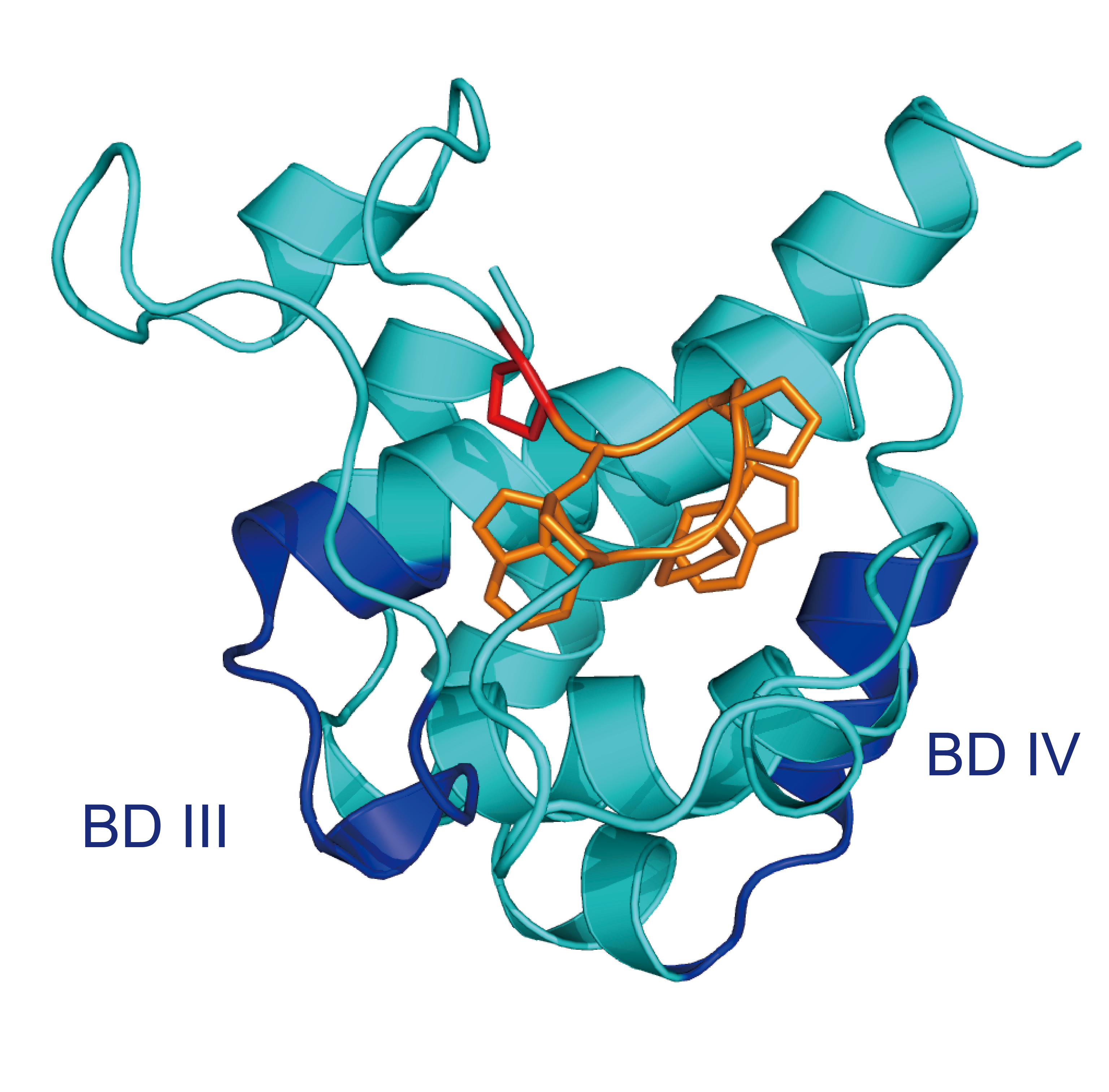

Supplement: S11 Fig — BD III and BD IV are in dark blue; “PPPWWP” (211–216) is in orange (P216 is marked by red). (TIF) [file pone.0137439.s011.tif]
