# Supplementary material for: Biochemical and Structural Insights into the Mechanism of DNA Recognition by Arabidopsis ETHYLENE INSENSITIVE3
Source: PLoS One. 2015 Sep 9;10(9):e0137439. doi: 10.1371/journal.pone.0137439 (PMC4564277; doi:10.1371/journal.pone.0137439)
Supplement: S1 File — (PDF) [file pone.0137439.s012.pdf]

Mascot Search Results

Protein View

Match to: **IPI00540843** Score: **4155**
Tax_Id=3702 Gene_Symbol=EIN3 Protein ETHYLENE INSENSITIVE 3
Found in search of D:\Phoenix\14-096_SJH0425_1_140506063512.mgf

Nominal mass (M_r): **72003**; Calculated pI value: **5.62**
NCBI BLAST search of IPI00540843 against nr
Unformatted sequence string for pasting into other applications

Fixed modifications: Carbamidomethyl (C)
Variable modifications: Oxidation (M)
No enzyme cleavage specificity
Sequence Coverage: **37%**

Matched peptides shown in **Bold Red**

```
  1 MMFNEMGMCG NMDFFSSGSL GEVDFCPVPQ AEPDSIVEDD YTDDEIDVDE
 51 LERRMWRDKM RLKRLKEQDK GKEGVDAAKQ RQSQEQARRK KMSRAQDGIL
101 KYMLKMMEVC KAQGFVYGII PENGKPVTGA SDNLREWWKD KVRFDRNGPA
151 AITKYQAENN IPGIHEGNNP IGPTPHTLQE LQDTTLGSLL SALMQHCDPP
201 QRRFPLEKGV PPPWWPNGKE DWWPQLGLPK DQGPAPYKKP HDLKKAWKVG
251 VLTAVIKHMF PDIAKIRKLV RQSKCLQDKM TAKESATWLA IINQEESLAR
301 ELYPESCPPL SLSGGSCSLL MNDCSQYDVE GFEKESHYEV EELKPEKVMN
351 SSNFGMVAKM HDFPVKEEVP AGNSEFMRKR KPNRDLNTIM DRTVFTCENL
401 GCAHSEISRG FLDRNSRDNH QLACPHRDSR LPYGAAPSRF HVNEVKPVVG
451 FPQPRPVNSV AQPIDLTGIV PEDGQKMISE LMSMYDRNVQ SNQTSMVMEN
501 QSVSLLQPTV HNHQEHLQFP GNMVEGSFFE DLNIPNRANN NNSSNNQTFF
551 QGNNNNNNVF KFDTADHNNF EAAHNNNNNS SGNRFQLVFD STPFDMASFD
601 YRDDMSMPGV VGTMDGMQQK QQDVSIWF
```

Sort Peptides By ◉ Residue Number ○ Increasing Mass ○ Decreasing Mass

Start - End	Observed	Mr(expt)	Mr(calc)	ppm	Miss	Sequence
95 - 105	640.3544	1278.6942	1278.7006	-5	0	R.AQDGILKYMLK.M (Ions score 58)
102 - 111	666.8104	1331.6062	1331.6110	-4	0	K.YMLKMMEVCK.A (Ions score 30)
102 - 111	674.8129	1347.6113	1347.6059	4	0	K.YMLKMMEVCK.A Oxidation (M) (Ions score 40)
106 - 111	415.1661	828.3177	828.3180	-0	0	K.MMEVCK.A 2 Oxidation (M) (Ions score 37)
106 - 135	1094.5295	3280.5668	3280.6043	-11	0	K.MMEVCKAQGFVYGIIPENGKPVTGASDNLR.E (Ions score 53)
112 - 125	746.8899	1491.7652	1491.7722	-5	0	K.AQGFVYGIIPENGK.P (Ions score 46)
112 - 131	669.0138	2004.0196	2004.0316	-6	0	K.AQGFVYGIIPENGKPVTGAS.D (Ions score 35)
112 - 131	1003.0219	2004.0293	2004.0316	-1	0	K.AQGFVYGIIPENGKPVTGAS.D (Ions score 47)
112 - 135	835.0980	2502.2721	2502.2867	-6	0	K.AQGFVYGIIPENGKPVTGASDNLR.E (Ions score 48)
112 - 135	835.0988	2502.2746	2502.2867	-5	0	K.AQGFVYGIIPENGKPVTGASDNLR.E (Ions score 61)
112 - 139	1044.8635	3131.5687	3131.5829	-5	0	K.AQGFVYGIIPENGKPVTGASDNLREWWK.D (Ions score 53)
112 - 141	1125.9021	3374.6845	3374.7048	-6	0	K.AQGFVYGIIPENGKPVTGASDNLREWWKDK.V (Ions score 41)
118 - 135	613.3290	1836.9651	1836.9694	-2	0	Y.GIIPENGKPVTGASDNLR.E (Ions score 29)
126 - 139	553.6107	1657.8101	1657.8212	-7	0	K.PVTGASDNLREWWK.D (Ions score 31)
132 - 139	573.7851	1145.5556	1145.5618	-5	0	S.DNLREWWK.D (Ions score 28)
147 - 154	386.2215	770.4285	770.4286	-0	0	R.NGPAAITK.Y (Ions score 59)
147 - 154	386.2217	770.4289	770.4286	0	0	R.NGPAAITK.Y (Ions score 57)
147 - 154	386.2220	770.4295	770.4286	1	0	R.NGPAAITK.Y (Ions score 33)
154 - 182	1070.5353	3208.5840	3208.5901	-2	0	T.KYQAENNIPGIHEGNNPIGPTPHTLQELQ.D (Ions score 57)
155 - 165	628.3067	1254.5989	1254.5993	-0	0	K.YQAENNIPGIH.E (Ions score 55)
155 - 165	628.3074	1254.6002	1254.5993	1	0	K.YQAENNIPGIH.E (Ions score 42)
155 - 167	721.3373	1440.6601	1440.6633	-2	0	K.YQAENNIPGIHEG.N (Ions score 57)
155 - 167	721.3386	1440.6626	1440.6633	-1	0	K.YQAENNIPGIHEG.N (Ions score 43)
155 - 168	778.3600	1554.7054	1554.7063	-1	0	K.YQAENNIPGIHEGN.N (Ions score 47)
155 - 168	778.3604	1554.7063	1554.7063	0	0	K.YQAENNIPGIHEGN.N (Ions score 78)
155 - 168	778.3609	1554.7073	1554.7063	1	0	K.YQAENNIPGIHEGN.N (Ions score 43)
155 - 169	835.3815	1668.7485	1668.7492	-0	0	K.YQAENNIPGIHEGNN.P (Ions score 43)
155 - 169	835.3819	1668.7492	1668.7492	0	0	K.YQAENNIPGIHEGNN.P (Ions score 44)
155 - 169	835.3829	1668.7512	1668.7492	1	0	K.YQAENNIPGIHEGNN.P (Ions score 59)
155 - 176	790.3747	2368.1023	2368.1196	-7	0	K.YQAENNIPGIHEGNNPIGPTPH.T (Ions score 61)
155 - 176	790.3748	2368.1026	2368.1196	-7	0	K.YQAENNIPGIHEGNNPIGPTPH.T (Ions score 59)
155 - 176	790.3763	2368.1070	2368.1196	-5	0	K.YQAENNIPGIHEGNNPIGPTPH.T (Ions score 32)
155 - 176	790.3768	2368.1087	2368.1196	-5	0	K.YQAENNIPGIHEGNNPIGPTPH.T (Ions score 55)
155 - 176	790.3784	2368.1132	2368.1196	-3	0	K.YQAENNIPGIHEGNNPIGPTPH.T (Ions score 45)
155 - 176	790.3807	2368.1202	2368.1196	0	0	K.YQAENNIPGIHEGNNPIGPTPH.T (Ions score 56)
155 - 178	861.7545	2582.2415	2582.2513	-4	0	K.YQAENNIPGIHEGNNPIGPTPHTL.Q (Ions score 48)
155 - 179	1356.1503	2710.2860	2710.3099	-9	0	K.YQAENNIPGIHEGNNPIGPTPHTLQ.E (Ions score 29)
155 - 179	904.4371	2710.2894	2710.3099	-8	0	K.YQAENNIPGIHEGNNPIGPTPHTLQ.E (Ions score 33)
155 - 179	904.4384	2710.2934	2710.3099	-6	0	K.YQAENNIPGIHEGNNPIGPTPHTLQ.E (Ions score 51)
155 - 179	904.4391	2710.2954	2710.3099	-5	0	K.YQAENNIPGIHEGNNPIGPTPHTLQ.E (Ions score 43)
155 - 182	1027.8307	3080.4702	3080.4951	-8	0	K.YQAENNIPGIHEGNNPIGPTPHTLQELQ.D (Ions score 55)
155 - 182	1541.2446	3080.4747	3080.4951	-7	0	K.YQAENNIPGIHEGNNPIGPTPHTLQELQ.D (Ions score 40)
155 - 182	1027.8333	3080.4779	3080.4951	-6	0	K.YQAENNIPGIHEGNNPIGPTPHTLQELQ.D (Ions score 47)
155 - 182	1027.8348	3080.4827	3080.4951	-4	0	K.YQAENNIPGIHEGNNPIGPTPHTLQELQ.D (Ions score 48)
155 - 182	1027.8350	3080.4831	3080.4951	-4	0	K.YQAENNIPGIHEGNNPIGPTPHTLQELQ.D (Ions score 52)
155 - 182	1027.8356	3080.4849	3080.4951	-3	0	K.YQAENNIPGIHEGNNPIGPTPHTLQELQ.D (Ions score 54)
155 - 182	1027.8359	3080.4860	3080.4951	-3	0	K.YQAENNIPGIHEGNNPIGPTPHTLQELQ.D (Ions score 41)
155 - 197	1570.4215	4708.2427	4708.2650	-5	0	K.YQAENNIPGIHEGNNPIGPTPHTLQELQDTTLGSLLSALMQHC.D (Ions score 52)
155 - 197	1575.7732	4724.2978	4724.2599	8	0	K.YQAENNIPGIHEGNNPIGPTPHTLQELQDTTLGSLLSALMQHC.D Oxidation (M) (Ions score 41)
157 - 176	1039.5038	2076.9930	2076.9977	-2	0	Q.AENNIPGIHEGNNPIGPTPH.T (Ions score 73)
158 - 176	669.6614	2005.9623	2005.9606	1	0	A.ENNIPGIHEGNNPIGPTPH.T (Ions score 57)
158 - 182	907.1129	2718.3167	2718.3361	-7	0	A.ENNIPGIHEGNNPIGPTPHTLQELQ.D (Ions score 38)
160 - 182	826.0846	2475.2320	2475.2506	-8	0	N.NIPGIHEGNNPIGPTPHTLQELQ.D (Ions score 35)
161 - 176	550.6171	1648.8294	1648.8322	-2	0	N.IPGIHEGNNPIGPTPH.T (Ions score 27)
162 - 176	768.8762	1535.7378	1535.7481	-7	0	I.PGIHEGNNPIGPTPH.T (Ions score 86)
166 - 178	673.8386	1345.6627	1345.6626	0	0	H.EGNNPIGPTPHTL.Q (Ions score 33)
166 - 179	737.8676	1473.7206	1473.7212	-0	0	H.EGNNPIGPTPHTLQ.E (Ions score 39)
166 - 179	737.8685	1473.7225	1473.7212	1	0	H.EGNNPIGPTPHTLQ.E (Ions score 33)
166 - 182	922.9565	1843.8984	1843.9064	-4	0	H.EGNNPIGPTPHTLQELQ.D (Ions score 52)
167 - 182	858.4332	1714.8518	1714.8638	-7	0	E.GNNPIGPTPHTLQELQ.D (Ions score 38)
169 - 182	772.9017	1543.7889	1543.7995	-7	0	N.NPIGPTPHTLQELQ.D (Ions score 39)
169 - 182	772.9030	1543.7915	1543.7995	-5	0	N.NPIGPTPHTLQELQ.D (Ions score 49)
170 - 182	715.8807	1429.7468	1429.7565	-7	0	N.PIGPTPHTLQELQ.D (Ions score 62)
170 - 182	715.8809	1429.7472	1429.7565	-7	0	N.PIGPTPHTLQELQ.D (Ions score 62)
177 - 202	984.8182	2951.4329	2951.4481	-5	0	H.TLQELQDTTLGSLLSALMQHCDPPQR.R (Ions score 65)
177 - 202	984.8206	2951.4400	2951.4481	-3	0	H.TLQELQDTTLGSLLSALMQHCDPPQR.R (Ions score 64)
177 - 202	990.1503	2967.4292	2967.4430	-5	0	H.TLQELQDTTLGSLLSALMQHCDPPQR.R Oxidation (M) (Ions score 55)
177 - 203	1036.8574	3107.5504	3107.5492	0	0	H.TLQELQDTTLGSLLSALMQHCDPPQRR.F (Ions score 30)
180 - 197	1008.9830	2015.9514	2015.9656	-7	0	Q.ELQDTTLGSLLSALMQHC.D (Ions score 123)
183 - 194	619.3154	1236.6163	1236.6272	-9	0	Q.DTTLGSLLSALM.Q Oxidation (M) (Ions score 63)
183 - 196	743.8781	1485.7416	1485.7497	-6	0	Q.DTTLGSLLSALMQH.C (Ions score 107)
183 - 196	751.8773	1501.7400	1501.7446	-3	0	Q.DTTLGSLLSALMQH.C Oxidation (M) (Ions score 64)
183 - 196	751.8793	1501.7441	1501.7446	-0	0	Q.DTTLGSLLSALMQH.C Oxidation (M) (Ions score 28)

183 - 197	823.8902	1645.7658	1645.7804	-9	0	Q.DTTLGSLLSALMQHC.D		(Ions score 57)
183 - 197	823.8929	1645.7712	1645.7804	-6	0	Q.DTTLGSLLSALMQHC.D		(Ions score 93)
183 - 197	823.8963	1645.7780	1645.7804	-1	0	Q.DTTLGSLLSALMQHC.D		(Ions score 56)
183 - 197	831.8895	1661.7645	1661.7753	-6	0	Q.DTTLGSLLSALMQHC.D	Oxidation (M)	(Ions score 52)
183 - 197	831.8896	1661.7647	1661.7753	-6	0	Q.DTTLGSLLSALMQHC.D	Oxidation (M)	(Ions score 56)
183 - 197	831.8900	1661.7655	1661.7753	-6	0	Q.DTTLGSLLSALMQHC.D	Oxidation (M)	(Ions score 89)
183 - 197	831.8913	1661.7680	1661.7753	-4	0	Q.DTTLGSLLSALMQHC.D	Oxidation (M)	(Ions score 105)
183 - 197	831.8915	1661.7685	1661.7753	-4	0	Q.DTTLGSLLSALMQHC.D	Oxidation (M)	(Ions score 96)
183 - 197	831.8920	1661.7694	1661.7753	-4	0	Q.DTTLGSLLSALMQHC.D	Oxidation (M)	(Ions score 102)
183 - 197	831.8928	1661.7711	1661.7753	-3	0	Q.DTTLGSLLSALMQHC.D	Oxidation (M)	(Ions score 81)
183 - 197	831.8936	1661.7727	1661.7753	-2	0	Q.DTTLGSLLSALMQHC.D	Oxidation (M)	(Ions score 44)
183 - 198	881.4061	1760.7977	1760.8073	-5	0	Q.DTTLGSLLSALMQHCD.P		(Ions score 89)
183 - 202	747.3595	2239.0567	2239.0725	-7	0	Q.DTTLGSLLSALMQHCDPPQR.R		(Ions score 40)
183 - 202	747.3607	2239.0603	2239.0725	-5	0	Q.DTTLGSLLSALMQHCDPPQR.R		(Ions score 66)
183 - 202	1120.5400	2239.0654	2239.0725	-3	0	Q.DTTLGSLLSALMQHCDPPQR.R		(Ions score 51)
184 - 197	766.3817	1530.7488	1530.7534	-3	0	D.TTLGSLLSALMQHC.D		(Ions score 59)
187 - 202	905.4445	1808.8744	1808.8662	5	0	L.GSLLSALMQHCDPPQR.R		(Ions score 53)
203 - 208	395.2344	788.4543	788.4545	-0	0	R.RFPLEK.G		(Ions score 30)
203 - 208	395.2354	788.4563	788.4545	2	0	R.RFPLEK.G		(Ions score 29)
209 - 216	468.2411	934.4676	934.4701	-3	0	K.GVPPPWWP.N		(Ions score 29)
209 - 219	617.8177	1233.6208	1233.6295	-7	0	K.GVPPPWWPNGK.E		(Ions score 39)
209 - 219	617.8180	1233.6214	1233.6295	-7	0	K.GVPPPWWPNGK.E		(Ions score 67)
209 - 219	617.8190	1233.6235	1233.6295	-5	0	K.GVPPPWWPNGK.E		(Ions score 39)
209 - 220	682.3394	1362.6642	1362.6721	-6	0	K.GVPPPWWPNGKE.D		(Ions score 30)
211 - 219	539.7731	1077.5316	1077.5396	-7	0	V.PPPWWPNGK.E		(Ions score 50)
211 - 219	539.7731	1077.5317	1077.5396	-7	0	V.PPPWWPNGK.E		(Ions score 43)
211 - 219	539.7746	1077.5346	1077.5396	-5	0	V.PPPWWPNGK.E		(Ions score 51)
212 - 219	491.2498	980.4851	980.4868	-2	0	P.PPWWPNGK.E		(Ions score 39)
220 - 230	684.8458	1367.6771	1367.6874	-8	0	K.EDWWPQLGLPK.D		(Ions score 44)
220 - 230	684.8469	1367.6793	1367.6874	-6	0	K.EDWWPQLGLPK.D		(Ions score 59)
220 - 230	684.8470	1367.6794	1367.6874	-6	0	K.EDWWPQLGLPK.D		(Ions score 49)
220 - 230	684.8470	1367.6794	1367.6874	-6	0	K.EDWWPQLGLPK.D		(Ions score 55)
220 - 230	684.8471	1367.6797	1367.6874	-6	0	K.EDWWPQLGLPK.D		(Ions score 51)
220 - 230	684.8474	1367.6802	1367.6874	-5	0	K.EDWWPQLGLPK.D		(Ions score 60)
220 - 230	684.8475	1367.6804	1367.6874	-5	0	K.EDWWPQLGLPK.D		(Ions score 65)
220 - 230	684.8475	1367.6805	1367.6874	-5	0	K.EDWWPQLGLPK.D		(Ions score 39)
220 - 230	684.8476	1367.6806	1367.6874	-5	0	K.EDWWPQLGLPK.D		(Ions score 54)
220 - 230	684.8483	1367.6821	1367.6874	-4	0	K.EDWWPQLGLPK.D		(Ions score 29)
220 - 230	684.8483	1367.6821	1367.6874	-4	0	K.EDWWPQLGLPK.D		(Ions score 39)
220 - 230	684.8488	1367.6830	1367.6874	-3	0	K.EDWWPQLGLPK.D		(Ions score 31)
220 - 230	684.8488	1367.6831	1367.6874	-3	0	K.EDWWPQLGLPK.D		(Ions score 36)
221 - 230	620.3250	1238.6354	1238.6448	-8	0	E.DWWPQLGLPK.D		(Ions score 37)
221 - 230	620.3264	1238.6383	1238.6448	-5	0	E.DWWPQLGLPK.D		(Ions score 57)
221 - 230	620.3266	1238.6386	1238.6448	-5	0	E.DWWPQLGLPK.D		(Ions score 48)
221 - 230	620.3271	1238.6396	1238.6448	-4	0	E.DWWPQLGLPK.D		(Ions score 34)
221 - 230	620.3276	1238.6406	1238.6448	-3	0	E.DWWPQLGLPK.D		(Ions score 30)
221 - 230	620.3278	1238.6410	1238.6448	-3	0	E.DWWPQLGLPK.D		(Ions score 42)
224 - 230	376.7369	751.4593	751.4592	0	0	W.PQLGLPK.D		(Ions score 36)
231 - 238	438.2162	874.4178	874.4185	-1	0	K.DQGPAPYK.K		(Ions score 57)
231 - 238	438.2165	874.4184	874.4185	-0	0	K.DQGPAPYK.K		(Ions score 33)
231 - 238	438.2169	874.4193	874.4185	1	0	K.DQGPAPYK.K		(Ions score 37)
239 - 245	433.2665	864.5184	864.5181	0	0	K.KPHDLKK.A		(Ions score 31)
245 - 257	471.6337	1411.8794	1411.8915	-9	0	K.KAWKVGVLTAVIK.H		(Ions score 60)
245 - 257	471.6343	1411.8812	1411.8915	-7	0	K.KAWKVGVLTAVIK.H		(Ions score 37)
245 - 257	471.6357	1411.8852	1411.8915	-4	0	K.KAWKVGVLTAVIK.H		(Ions score 48)
246 - 257	642.9003	1283.7860	1283.7965	-8	0	K.AWKVGVLTAVIK.H		(Ions score 49)
246 - 257	642.9015	1283.7884	1283.7965	-6	0	K.AWKVGVLTAVIK.H		(Ions score 55)
246 - 257	642.9060	1283.7975	1283.7965	1	0	K.AWKVGVLTAVIK.H		(Ions score 58)
249 - 257	450.2963	898.5780	898.5852	-8	0	K.VGVLTAVIK.H		(Ions score 65)
249 - 257	450.2966	898.5787	898.5852	-7	0	K.VGVLTAVIK.H		(Ions score 67)
249 - 257	450.2967	898.5789	898.5852	-7	0	K.VGVLTAVIK.H		(Ions score 70)
249 - 257	450.2971	898.5797	898.5852	-6	0	K.VGVLTAVIK.H		(Ions score 51)
249 - 257	450.2971	898.5797	898.5852	-6	0	K.VGVLTAVIK.H		(Ions score 69)
249 - 257	450.2972	898.5799	898.5852	-6	0	K.VGVLTAVIK.H		(Ions score 70)
249 - 257	450.2975	898.5805	898.5852	-5	0	K.VGVLTAVIK.H		(Ions score 68)
249 - 257	450.2981	898.5816	898.5852	-4	0	K.VGVLTAVIK.H		(Ions score 65)
249 - 257	450.2992	898.5838	898.5852	-2	0	K.VGVLTAVIK.H		(Ions score 69)
258 - 265	479.7431	957.4717	957.4742	-3	0	K.HMFPDIAK.I		(Ions score 64)
258 - 265	479.7438	957.4730	957.4742	-1	0	K.HMFPDIAK.I		(Ions score 50)
258 - 265	479.7440	957.4734	957.4742	-1	0	K.HMFPDIAK.I		(Ions score 60)
258 - 265	479.7443	957.4740	957.4742	-0	0	K.HMFPDIAK.I		(Ions score 46)
258 - 265	479.7447	957.4748	957.4742	1	0	K.HMFPDIAK.I		(Ions score 40)
258 - 265	487.7411	973.4676	973.4691	-2	0	K.HMFPDIAK.I	Oxidation (M)	(Ions score 49)
258 - 265	487.7418	973.4690	973.4691	-0	0	K.HMFPDIAK.I	Oxidation (M)	(Ions score 49)
258 - 265	487.7419	973.4692	973.4691	0	0	K.HMFPDIAK.I	Oxidation (M)	(Ions score 41)
258 - 265	487.7419	973.4693	973.4691	0	0	K.HMFPDIAK.I	Oxidation (M)	(Ions score 37)
258 - 265	487.7420	973.4695	973.4691	0	0	K.HMFPDIAK.I	Oxidation (M)	(Ions score 44)
258 - 265	487.7420	973.4695	973.4691	0	0	K.HMFPDIAK.I	Oxidation (M)	(Ions score 50)
258 - 265	487.7421	973.4696	973.4691	1	0	K.HMFPDIAK.I	Oxidation (M)	(Ions score 35)
258 - 265	487.7421	973.4697	973.4691	1	0	K.HMFPDIAK.I	Oxidation (M)	(Ions score 43)
258 - 265	487.7422	973.4699	973.4691	1	0	K.HMFPDIAK.I	Oxidation (M)	(Ions score 42)
258 - 265	487.7422	973.4699	973.4691	1	0	K.HMFPDIAK.I	Oxidation (M)	(Ions score 36)
258 - 265	487.7424	973.4702	973.4691	1	0	K.HMFPDIAK.I	Oxidation (M)	(Ions score 29)
258 - 265	487.7447	973.4748	973.4691	6	0	K.HMFPDIAK.I	Oxidation (M)	(Ions score 35)
258 - 265	487.7458	973.4770	973.4691	8	0	K.HMFPDIAK.I	Oxidation (M)	(Ions score 30)
258 - 267	409.8940	1226.6603	1226.6594	1	0	K.HMFPDIAKIR.K		(Ions score 53)
284 - 300	965.9888	1929.9631	1929.9796	-9	0	K.ESATWLAIINQEESLAR.E		(Ions score 90)
284 - 300	965.9897	1929.9648	1929.9796	-8	0	K.ESATWLAIINQEESLAR.E		(Ions score 82)
284 - 300	965.9908	1929.9670	1929.9796	-6	0	K.ESATWLAIINQEESLAR.E		(Ions score 97)
284 - 300	965.9911	1929.9677	1929.9796	-6	0	K.ESATWLAIINQEESLAR.E		(Ions score 80)
284 - 300	965.9913	1929.9681	1929.9796	-6	0	K.ESATWLAIINQEESLAR.E		(Ions score 73)
284 - 300	965.9918	1929.9690	1929.9796	-5	0	K.ESATWLAIINQEESLAR.E		(Ions score 102)
284 - 300	965.9918	1929.9691	1929.9796	-5	0	K.ESATWLAIINQEESLAR.E		(Ions score 80)
284 - 300	965.9920	1929.9695	1929.9796	-5	0	K.ESATWLAIINQEESLAR.E		(Ions score 81)
284 - 300	965.9921	1929.9696	1929.9796	-5	0	K.ESATWLAIINQEESLAR.E		(Ions score 73)
284 - 300	965.9926	1929.9707	1929.9796	-5	0	K.ESATWLAIINQEESLAR.E		(Ions score 85)
284 - 300	965.9927	1929.9708	1929.9796	-5	0	K.ESATWLAIINQEESLAR.E		(Ions score 97)
284 - 300	644.3313	1929.9721	1929.9796	-4	0	K.ESATWLAIINQEESLAR.E		(Ions score 66)
284 - 300	965.9933	1929.9721	1929.9796	-4	0	K.ESATWLAIINQEESLAR.E		(Ions score 83)
284 - 300	965.9934	1929.9723	1929.9796	-4	0	K.ESATWLAIINQEESLAR.E		(Ions score 106)
284 - 300	965.9949	1929.9753	1929.9796	-2	0	K.ESATWLAIINQEESLAR.E		(Ions score 84)
284 - 300	965.9952	1929.9758	1929.9796	-2	0	K.ESATWLAIINQEESLAR.E		(Ions score 81)
284 - 300	965.9965	1929.9784	1929.9796	-1	0	K.ESATWLAIINQEESLAR.E		(Ions score 81)
288 - 300	771.9122	1541.8099	1541.8201	-7	0	T.WLAIINQEESLAR.E		(Ions score 57)
289 - 300	678.8676	1355.7206	1355.7408	-15	0	W.LAIINQEESLAR.E		(Ions score 85)
289 - 300	678.8742	1355.7339	1355.7408	-5	0	W.LAIINQEESLAR.E		(Ions score 105)
290 - 300	622.3351	1242.6556	1242.6568	-1	0	L.AIINQEESLAR.E		(Ions score 59)
290 - 300	622.3354	1242.6563	1242.6568	-0	0	L.AIINQEESLAR.E		(Ions score 71)
290 - 300	622.3356	1242.6567	1242.6568	-0	0	L.AIINQEESLAR.E		(Ions score 55)
301 - 316	846.8901	1691.7657	1691.7712	-3	0	R.ELYPESCPPLSLSGGS.C		(Ions score 35)
301 - 322	1206.0433	2410.0721	2410.0854	-6	0	R.ELYPESCPPLSLSGGSCSLLMN.D		(Ions score 36)
301 - 322	1214.0438	2426.0731	2426.0804	-3	0	R.ELYPESCPPLSLSGGSCSLLMN.D	Oxidation (M)	(Ions score 39)

301 - 327	1022.1027	3063.2862	3063.2970	-4	0	R.ELYPESCPPLSLSGGSCSLLMNDCSQY.D	(Ions score 57)
323 - 334	738.7968	1475.5790	1475.5875	-6	0	N.DCSQYDVEGFEK.E	(Ions score 39)
323 - 334	738.7989	1475.5832	1475.5875	-3	0	N.DCSQYDVEGFEK.E	(Ions score 33)
323 - 334	738.8000	1475.5855	1475.5875	-1	0	N.DCSQYDVEGFEK.E	(Ions score 67)
323 - 334	738.8004	1475.5862	1475.5875	-1	0	N.DCSQYDVEGFEK.E	(Ions score 53)
323 - 334	738.8042	1475.5938	1475.5875	4	0	N.DCSQYDVEGFEK.E	(Ions score 65)
323 - 347	1025.4528	3073.3364	3073.3498	-4	0	N.DCSQYDVEGFEKESHYEVEELKPEK.V	(Ions score 56)
324 - 334	681.2872	1360.5599	1360.5605	-0	0	D.CSQYDVEGFEK.E	(Ions score 43)
325 - 334	601.2694	1200.5243	1200.5299	-5	0	C.SQYDVEGFEK.E	(Ions score 49)
328 - 334	412.1951	822.3757	822.3759	-0	0	Y.DVEGFEK.E	(Ions score 29)
328 - 338	670.2938	1338.5731	1338.5728	0	0	Y.DVEGFEKESHY.E	(Ions score 36)
328 - 341	848.8638	1695.7131	1695.7264	-8	0	Y.DVEGFEKESHYEVE.E	(Ions score 35)
328 - 341	848.8657	1695.7169	1695.7264	-6	0	Y.DVEGFEKESHYEVE.E	(Ions score 44)
328 - 341	848.8661	1695.7176	1695.7264	-5	0	Y.DVEGFEKESHYEVE.E	(Ions score 40)
328 - 341	566.2470	1695.7192	1695.7264	-4	0	Y.DVEGFEKESHYEVE.E	(Ions score 27)
328 - 341	848.8675	1695.7204	1695.7264	-4	0	Y.DVEGFEKESHYEVE.E	(Ions score 28)
328 - 341	848.8677	1695.7208	1695.7264	-3	0	Y.DVEGFEKESHYEVE.E	(Ions score 40)
328 - 341	848.8677	1695.7209	1695.7264	-3	0	Y.DVEGFEKESHYEVE.E	(Ions score 48)
328 - 341	848.8696	1695.7247	1695.7264	-1	0	Y.DVEGFEKESHYEVE.E	(Ions score 53)
328 - 341	848.8698	1695.7249	1695.7264	-1	0	Y.DVEGFEKESHYEVE.E	(Ions score 54)
328 - 341	848.8721	1695.7297	1695.7264	2	0	Y.DVEGFEKESHYEVE.E	(Ions score 40)
328 - 347	807.7155	2420.1245	2420.1383	-6	0	Y.DVEGFEKESHYEVEELKPEK.V	(Ions score 41)
328 - 347	807.7160	2420.1262	2420.1383	-5	0	Y.DVEGFEKESHYEVEELKPEK.V	(Ions score 44)
328 - 347	807.7161	2420.1265	2420.1383	-5	0	Y.DVEGFEKESHYEVEELKPEK.V	(Ions score 42)
328 - 347	807.7162	2420.1269	2420.1383	-5	0	Y.DVEGFEKESHYEVEELKPEK.V	(Ions score 43)
328 - 347	807.7189	2420.1348	2420.1383	-1	0	Y.DVEGFEKESHYEVEELKPEK.V	(Ions score 45)
328 - 347	807.7191	2420.1353	2420.1383	-1	0	Y.DVEGFEKESHYEVEELKPEK.V	(Ions score 44)
328 - 347	807.7197	2420.1372	2420.1383	-0	0	Y.DVEGFEKESHYEVEELKPEK.V	(Ions score 41)
328 - 347	807.7205	2420.1397	2420.1383	1	0	Y.DVEGFEKESHYEVEELKPEK.V	(Ions score 41)
330 - 341	741.8196	1481.6246	1481.6310	-4	0	V.EGFEKESHYEVE.E	(Ions score 33)
335 - 347	539.5975	1615.7706	1615.7729	-1	0	K.ESHYEVEELKPEK.V	(Ions score 28)
335 - 347	539.5981	1615.7724	1615.7729	-0	0	K.ESHYEVEELKPEK.V	(Ions score 37)
335 - 347	539.5984	1615.7735	1615.7729	0	0	K.ESHYEVEELKPEK.V	(Ions score 39)
335 - 347	539.5990	1615.7752	1615.7729	1	0	K.ESHYEVEELKPEK.V	(Ions score 30)
335 - 352	712.3337	2133.9792	2133.9888	-4	0	K.ESHYEVEELKPEKVMNSS.N	(Ions score 30)

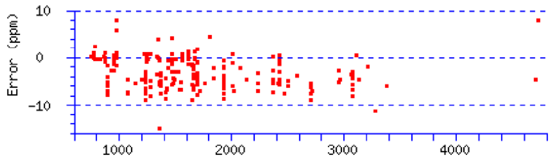

RMS error 4 ppm

```
ID   IPI00540843.1         IPI;      PRT;   628 AA.
AC   IPI00540843;
DT   05-JAN-2005 (IPI Arabidopsis rel. 3.00, Created)
DT   05-JAN-2005 (IPI Arabidopsis rel. 3.00, Last sequence update)
DE   PROTEIN ETHYLENE INSENSITIVE 3.
OS   Arabidopsis thaliana (Mouse-ear cress).
OC   Eukaryota; Viridiplantae; Streptophyta; Embryophyta; Tracheophyta;
OC   Spermatophyta; Magnoliophyta; eudicotyledons; core eudicots; rosids;
OC   eurosids II; Brassicales; Brassicaceae; Arabidopsis.
OX   NCBI_TaxID=3702;
CC   -!- GENE_LOCATION: Chr. 3:7260702-7262588:-1.
DR   UniProtKB/Swiss-Prot; O24606; EIN3_ARATH; M.
DR   REFSEQ_REVIEWED; NP_188713; GI:15232362; -.
DR   TAIR Protein; AT3G20770.1; AT3G20770.1; -.
DR   UniProtKB/TrEMBL; B0LZR6; B0LZR6_ARATH; -.
DR   UniProtKB/TrEMBL; B0LZR7; B0LZR7_ARATH; -.
DR   UniProtKB/TrEMBL; B0LZR8; B0LZR8_ARATH; -.
DR   UniProtKB/TrEMBL; B0LZR9; B0LZR9_ARATH; -.
DR   UniProtKB/TrEMBL; B0LZS0; B0LZS0_ARATH; -.
DR   UniProtKB/TrEMBL; B0LZS2; B0LZS2_ARATH; -.
DR   UniProtKB/TrEMBL; B0LZS3; B0LZS3_ARATH; -.
DR   UniProtKB/TrEMBL; B0LZS6; B0LZS6_ARATH; -.
DR   UniProtKB/TrEMBL; B0LZT0; B0LZT0_ARATH; -.
DR   UniProtKB/TrEMBL; B0LZT2; B0LZT2_ARATH; -.
DR   UniProtKB/TrEMBL; B0LZT4; B0LZT4_ARATH; -.
DR   UniProtKB/TrEMBL; B0LZT7; B0LZT7_ARATH; -.
DR   UniProtKB/TrEMBL; B0LZT9; B0LZT9_ARATH; -.
DR   UniProtKB/TrEMBL; B0LZU2; B0LZU2_ARATH; -.
DR   UniProtKB/TrEMBL; B0LZU8; B0LZU8_ARATH; -.
DR   UniProtKB/TrEMBL; B0LZV1; B0LZV1_ARATH; -.
DR   UniProtKB/TrEMBL; B0LZV4; B0LZV4_ARATH; -.
DR   UniProtKB/TrEMBL; B0LZV5; B0LZV5_ARATH; -.
DR   UniProtKB/TrEMBL; B0LZV7; B0LZV7_ARATH; -.
DR   UniProtKB/TrEMBL; B0LZV9; B0LZV9_ARATH; -.
DR   UniProtKB/TrEMBL; B0LZW6; B0LZW6_ARATH; -.
DR   UniProtKB/TrEMBL; B0LZX3; B0LZX3_ARATH; -.
DR   UniProtKB/TrEMBL; B0LZX6; B0LZX6_ARATH; -.
DR   UniProtKB/TrEMBL; B0LZY5; B0LZY5_ARATH; -.
DR   UniProtKB/TrEMBL; B0LZY8; B0LZY8_ARATH; -.
DR   UniProtKB/TrEMBL; B0LZZ1; B0LZZ1_ARATH; -.
DR   UniProtKB/TrEMBL; B0LZZ3; B0LZZ3_ARATH; -.
DR   UniProtKB/TrEMBL; B0LZZ4; B0LZZ4_ARATH; -.
DR   UniProtKB/TrEMBL; B0LZZ5; B0LZZ5_ARATH; -.
DR   UniProtKB/TrEMBL; B0LZZ7; B0LZZ7_ARATH; -.
DR   UniProtKB/TrEMBL; B0LZZ8; B0LZZ8_ARATH; -.
DR   UniProtKB/TrEMBL; B0M000; B0M000_ARATH; -.
DR   UniProtKB/TrEMBL; B0M001; B0M001_ARATH; -.
DR   UniProtKB/TrEMBL; B0M003; B0M003_ARATH; -.
DR   UniProtKB/TrEMBL; B0M006; B0M006_ARATH; -.
DR   UniProtKB/TrEMBL; B0M007; B0M007_ARATH; -.
DR   UniParc; UPI000009EB88; -; -.
DR   TAIR Gene; AT3G20770; EIN3; -.
DR   Entrez Gene; 821625; EIN3; -.
DR   UniGene; At.24824; -; -.
DR   InterPro; IPR006957; EIN3.
DR   InterPro; IPR023278; Ethylene_insens-like_DNA-bd.
DR   Pfam; PF04873; EIN3; 1.
DR   SUPERFAMILY; SSF116768; SSF116768; 1.
DR   GENE3D; G3DSA:1.10.3180.10; Ethylene_insens_like_DNA_bd; 1.
SQ   SEQUENCE   628 AA;  71421 MW;  8DA269C9F2A3BFD3 CRC64;
     MMFNEMGMCG NMDFFSSGSL GEVDFCPVPQ AEPDSIVEDD YTDDEIDVDE LERRMWRDKM
     RLKRLKEQDK GKEGVDAAKQ RQSQEQARRK KMSRAQDGIL KYMLKMMEVC KAQGFVYGII
     PENGKPVTGA SDNLREWWKD KVRFDRNGPA AITKYQAENN IPGIHEGNNP IGPTPHTLQE
     LQDTTLGSLL SALMQHCDPP QRRFPLEKGV PPPWWPNGKE DWWPQLGLPK DQGPAPYKKP
```
